# Spatial architecture of atherosclerotic plaques: coordinating immune responses through mechanotransduction and vesicular trafficking

**DOI:** 10.3389/fcell.2026.1844243

**Published:** 2026-08-03

**Authors:** Xiaoyu Yu, Cong Cui, Jiyong He, Xiaoyu Guan, Zhe Zhang

**Affiliations:** 1 The Affiliated Hospital of Liaoning University of Traditional Chinese Medicine, Shenyang, China; 2 Liaoning University of Traditional Chinese Medicine, Shenyang, China

**Keywords:** atherosclerosis, efferocytosis, extracellular vesicles, immune microenvironment, mechanotransduction, Piezo1, plaque spatial architecture, YAP/TAZ

## Abstract

Atherosclerosis is a progressive inflammatory vascular disease, and its acute clinical consequences—myocardial infarction and ischemic stroke—are the principal cause of cardiovascular mortality globally. Plaque rupture, driven by dysregulated immune activity within the plaque microenvironment, is the immediate precipitant of acute cardiovascular events. Single-cell RNA sequencing, spatial transcriptomics, and high-resolution mechanobiology have shown that atherosclerotic plaques are spatially organised structures in which functionally divergent cell populations occupy the fibrous cap, necrotic core, shoulder region, and neovascularisation zone—a heterogeneity organised by two regulatory axes: mechanotransduction and vesicular trafficking. This review elucidates how the mechano-vesicular dual-axis shapes the plaque immune microenvironment and drives the transition from stable to vulnerable plaque phenotype, and assesses the translational potential of the framework for precision diagnostics and therapeutics. We here formally propose the Mechano-Vesicular Dual-Axis (MVDA) model as a unifying conceptual framework for plaque immune microenvironment organisation, defined by three constitutive elements: (i) a mechanotransduction axis converting hemodynamic and matrix-stiffness cues into intracellular signaling; (ii) a vesicular trafficking axis propagating mechanically-encoded information across cells; and (iii) bidirectional coupling through shared signaling nodes (KLF2/4, YAP/TAZ, NF-κB, PI3K/Akt/mTOR). To our knowledge, this is the first explicit integration of these two regulatory axes into a single testable framework for plaque spatial biology. Oscillatory shear stress and progressive matrix stiffening reprogramme endothelial cells, macrophages, and vascular smooth muscle cells through mechanosensors including Piezo1, integrins, and YAP/TAZ, driving pro-inflammatory gene programmes and spatially directed immune cell migration. Extracellular vesicles (EVs) generated under distinct mechanical stimuli carry bioactive cargo—including miR-155, miR-146a, oxidised phospholipids, and damage-associated molecular patterns—that establishes paracrine and long-range intercellular communication networks propagating mechanical activation signals. The two axes are connected through shared signaling nodes (KLF2/4 as an upstream flow-sensitive gate; YAP/TAZ and NF-κB as parallel effectors; PI3K/Akt/mTOR as vesicular output controllers). Dysregulation manifests as elevated pro-inflammatory EV output, macrophage and T cell accumulation at the shoulder region, impaired efferocytosis, and progressive fibrous cap thinning, marking the mechanistic transition toward the vulnerable plaque phenotype. The MVDA model suggests a move from systemic pharmacological intervention toward spatially precise targeting of mechanically high-risk microregions. Translational candidates include Piezo1 antagonists, YAP/TAZ inhibitors delivered via biomimetic nanoparticles, engineered M2 macrophage-derived EVs, and circulating EV-encapsulated miRNA panels as non-invasive biomarkers of plaque vulnerability. The MVDA model’s mechano-immune scaffold is substantially supported by human data; its vesicular reverse-coupling claims remain forward-looking and await *in vivo* genetic validation. Addressing methodological challenges—real-time mechanical signal quantification, standardised EV tracking, and the murine-to-human translational gap—will be central to the next phase of work.

## 1 Introduction

Atherosclerosis (AS) is a progressive vascular disease characterised by lipid deposition, chronic inflammation, and vascular remodeling. The acute myocardial infarction and ischemic stroke to which it gives rise are the leading causes of cardiovascular mortality worldwide (Roth et al., 2020). According to the Global Burden of Disease study, nearly 20 million deaths were attributable to cardiovascular disease—including atherosclerotic cardiovascular disease (ASCVD) — in 2022, accounting for approximately 32% of all-cause mortality, and this burden continues to grow with global population aging (Mensah et al., 2023). The clinical harm of atherosclerosis is not primarily attributable to progressive luminal narrowing in itself; it is plaque instability that matters. Rupture or erosion of the fibrous cap of vulnerable plaques triggers acute thrombosis and produces acute coronary syndromes (ACS) (Bentzon et al., 2014). Pathological investigations show that plaque rupture is the most common immediate precipitant of acute myocardial infarction, particularly in men, and that rupture-prone plaques frequently produce only moderate luminal stenosis (diameter stenosis <50%) before the acute event, making them largely undetectable by conventional anatomic imaging (Falk et al., 2013). The central pathological basis of plaque vulnerability is local immune dysregulation: excessive activation of pro-inflammatory macrophages, infiltration of effector T cells, impaired regulatory T cell (Treg) function, and defective apoptotic cell clearance (efferocytosis defect) together drive necrotic core expansion and fibrous cap thinning (Yurdagul et al., 2017; Wolf and Ley, 2019). Defining the molecular and cellular mechanisms that govern immune regulation within plaques is therefore a precondition for precision prevention and treatment of atherosclerosis.

For decades, atherosclerotic plaques were treated as relatively homogeneous lesions dominated by lipid deposition and chronic inflammation, and conventional research focused on circulating inflammatory biomarkers (e.g., C-reactive protein, interleukin-6) and bulk transcriptomic analyses of whole-plaque specimens (Wolf and Ley, 2019). This framing does not account for the markedly divergent functional states observed in cells from different regions of the same plaque, or for the highly focal nature of plaque vulnerability. Single-cell RNA sequencing (scRNA-seq) and related high-resolution omics technologies have changed how the internal organisation of plaques is understood. Multiple high-resolution single-cell atlas studies have profiled human carotid and coronary artery plaques and identified macrophage subpopulations with distinct functional characteristics—including pro-inflammatory (NLRP3^+^/IL-1β^+^), lipid-associated (TREM2^+^/CD9^+^), and proliferating subsets—that show non-random distribution patterns within plaques and are closely linked to smooth muscle cell (SMC) phenotypic switching (Depuydt et al., 2020; Mosquera et al., 2023; Pan et al., 2020). This refined spatial-functional compartmentalisation points to microenvironment-driven zonation mechanisms that operate above and beyond cell type identity, in which local physical signals and intercellular communication jointly determine the spatial order of the plaque (Pan et al., 2020). The implication is that strategies focused on single molecular targets or individual cell types are limited; understanding the spatial integrity of the plaque microenvironment is the necessary route to identifying actionable therapeutic targets.

Among the microenvironmental factors that shape spatial heterogeneity within plaques, mechanotransduction and vesicular trafficking are two coordinating mechanisms that have only recently received systematic attention. The local hemodynamic environment—particularly regions of oscillatory shear stress (OSS) and low shear stress—governs the anatomical predilection sites of atherosclerosis, and converts physical signals into pro-inflammatory gene expression programmes through Piezo1/2 mechanosensitive ion channels, the integrin–focal adhesion kinase (FAK) axis, and the YAP/TAZ transcriptional coactivators, supporting directional recruitment and functional polarisation of immune cells (Shinge et al., 2022). The dynamic elevation of matrix stiffness during plaque progression—from approximately 5–15 kPa in normal arterial walls to greater than 100 kPa in calcified plaques—remodels macrophage and SMC phenotypes through cytoskeletal tension (Wang Y. et al., 2022). Extracellular vesicles (EVs) within the plaque microenvironment (Hartman et al., 2016) serve as mediators of intercellular information transfer, carrying bioactive molecules including functional microRNAs, lipid antigens, and inflammatory proteins, and supporting paracrine communication networks among endothelial cells, macrophages, SMCs, and T cells (Nguyen et al., 2018). How mechanotransduction and vesicular trafficking together coordinate immune responses within the three-dimensional spatial architecture of plaques is the question this review addresses. the evidence-stratification convention used to assess its components is given in Box 2.

BOX 1 **Evidence stratification used throughout this review.**

We adopt a four-tier evidence framework, applied to every key mechanistic claim:

[E] Established mechanism—supported by multiple independent human studies and confirmed *in vivo* genetic loss/gain-of-function evidence.
[P] Strong preclinical evidence—robust murine or large-animal *in vivo* data with mechanistic intervention.
[I] Plausible inference—extrapolation from *in vitro* mechanistic studies or correlational human data without causal *in vivo* validation.
[S] Speculative extrapolation—forward-looking hypotheses advanced by this review that await empirical testing.

In particular, the mechano-vesicular reverse coupling (Box 2, C4) is currently supported principally by [I]-level evidence; direct *in vivo* genetic validation through endothelial-conditional Rab27a/b or Piezo1 knockouts combined with EV tracking is acknowledged as the principal outstanding requirement for graduating C4 from [I] to [E].

The MVDA model defined in Box 1 above is intended to capture how the mechanotransduction and vesicular trafficking axes together construct and maintain this spatial gradient. The four tiers are summarised in Table 2 and are appended to every key mechanistic claim. Against this background, the present review addresses how plaque spatial architecture coordinates immune responses through mechanotransduction and vesicular trafficking. The review is organised into seven substantive sections. Section 2 describes the spatial anatomical compartments, cellular distribution atlas, and physicochemical microenvironmental features of atherosclerotic plaques. Section 3 delineates the molecular mechanisms of mechanotransduction in the immune regulation of endothelial cells, macrophages, and SMCs. Section 4 covers the types, cargo, and functions of vesicular trafficking in intercellular immune communication within plaques. Section 5 proposes the integrated cross-regulatory network linking mechanotransduction and vesicular trafficking. Section 6 analyses the relationship between disrupted spatial architecture and plaque vulnerability from a mechanistic integration perspective and includes cross-validation against emerging human plaque spatial atlases. Section 7 discusses research frontiers, translational therapeutic candidates, and clinical readiness; Section 8 summarises outstanding challenges and future directions (Winkels et al., 2018; Bäck et al., 2019). We here formally propose the Mechano-Vesicular Dual-Axis (MVDA) model as a unifying conceptual framework for plaque immune microenvironment organisation. The MVDA model is defined by three constitutive elements: (i) a mechanotransduction axis converting hemodynamic and matrix-stiffness cues into intracellular signaling; (ii) a vesicular trafficking axis propagating mechanically-encoded information across cells; and (iii) bidirectional coupling through shared signaling nodes (KLF2/4, YAP/TAZ, NF-κB, PI3K/Akt/mTOR). The formal definition of the model is given in Box 2 (Section 5.3); the evidence-stratification convention used to assess its components is given in Box 1 above. To our knowledge, this is the first explicit integration of these two regulatory axes into a single, testable framework for plaque spatial biology.

**TABLE 1:** Evidence stratification of key mechanistic claims underlying the MVDA model.

#	Mechanistic claim	MVDA element	Tier	Key refs	Proposed validation/criterion for upgrade
1	Endothelial-specific Piezo1 knockout markedly reduces aortic KLF2/4, identifying the Piezo1–CaMKII–MEKK3–ERK5 axis as the link between shear sensing and KLF2/4 induction	C1	E	Zheng et al. (2022)	Already validated *in vivo*
2	Shear-stress-dependent differential EV cargo loading; integrin and Piezo1 packaging into EV membranes	C1→C2	E	Chung et al. (2022), Multhaupt et al. (2016), Mittelheisser et al. (2024)	Established
3	Piezo1 inhibition by GsMTx4 attenuates endothelial inflammation and delays plaque progression in ApoE^−/−^ mice (via Ca^2+^/CaMKII/FAK/Src and reduced YAP nuclear translocation)	C1	P	Lan et al. (2024)	Large-animal/human validation
4	Local stiffness gradients shape macrophage polarisation topology (soft zones favour M2, stiff zones favour M1), contributing to regional immune topology	C1, C5	P	—	*In situ* human spatial validation
5	Matrix stiffening and VSMC dedifferentiation sustain one another as atherosclerosis progresses	C1	P	Elmarasi et al. (2024)	Longitudinal human data
6	YAP/TAZ activity tunes MVB transport and exosome release via actin polymerisation and Rab-GTPase recruitment (mechanical input → EV output)	C3	P	Ritsvall and Albinsson (2024)	*In vivo* genetic validation
7	Endothelial exosomes carrying NF-κB-activating cargo engage NF-κB in recipient macrophages, forming a transcellular inflammatory amplification route	C2	P	Wang et al. (2023)	*In vivo* EV tracking
8	PI3K/Akt sets integrin display density on EVs and their recipient-cell targeting specificity	C3	P	Multhaupt et al. (2016)	*In vivo* validation
9	Exosomes from nicotine-stimulated macrophages are enriched in miR-21-3p (targets PTEN, activates PI3K/AKT), promoting VSMC migration/proliferation	C2	P	Zhu et al. (2019)	—
10	ExoM2 suppresses PDGF-BB-induced VSMC proliferation/migration and reduces plaque burden in ApoE^−/−^ mice	C2	P	Wang S. et al. (2024)	Human validation
11	Dual CD47/SIRPα + ANGPTL3 blockade enhances efferocytosis and attenuates plaque progression in animal models	C2	P	Hu et al. (2024)	Human validation
12	In vulnerable plaques, oxidative stress/hypoxia drive M1 and endothelial release of miR-155- and oxPL-enriched pro-inflammatory EVs	C2	P	Patel et al. (2023)	—
13	MMP-mediated ECM degradation and mechanical destabilisation drive one another reciprocally rather than sequentially	C1	P	—	*In vivo* temporal validation
14	Myeloid cells in ACS culprit plaques show stronger pro-inflammatory activation → immune-cell functional state, not only number, predicts vulnerability	C5	P	Emoto et al. (2022)	—
15	On stiffness-graded substrates, macrophages migrate preferentially toward stiffer zones (e.g., fibrous cap), concentrating pro-inflammatory cells in vulnerable regions	C1, C5	I	Shellard and Mayor (2021)	*In vivo*/human validation
16	OSS-induced endothelial Piezo1 activation raises permeability and couples to selective transendothelial immune infiltration	C1	I	Lan et al. (2024)	*In vivo* causal validation
17	Exosomal-membrane MHC-II presents antigen directly to CD4^+^ T cells without conventional APC intermediaries	C2	I	Buzas (2023)	*In vivo* validation
18	pEVs show spatial targeting advantage toward fibrin/collagen deposition, explored for plaque-targeted drug-delivery nanoplatforms	C2	I	Weiss et al. (2025), Ma et al. (2021)	Preclinical validation
19	Efferocytosis deficiency converts unresolved apoptotic load into pro-inflammatory vesicular output, sustaining local inflammatory amplification	C2	I	Wang Z. et al. (2022), Tall and Bornfeldt (2023)	*In vivo* validation
20	Circulating EV miRNAs (miR-193b-5p/193a-5p/125a-3p) are upregulated in carotid-stenosis patient serum with concordant plaque-tissue expression → circulating EVs reflect local pathology	Diagnostic	I	Brandes et al. (2024)	Prospective cohort validation
21	Mechano-vesicular reverse coupling (C4): EVs bearing integrins/Piezo1 from highly mechanically activated regions confer mechanical sensitivity on recipient cells in low-stimulus regions (“spatial permeation”)	C4	S (target E)	Mukhopadhyay et al. (2024)	Endothelial-conditional Rab27a/b or Piezo1 knockout + *in vivo* EV tracing (principal methodological dependency); human *ex vivo* plaque imaging-mass cytometry + EV phenotyping
22	A transferable “mechanical-state memory” propagated across vascular compartments	C4	S	—	Awaits *in vivo* genetic validation

Evidence tiers [E], [P], [I], and [S] are defined in Table 2. MVDA, model elements C1–C5 are defined in Box 1 (Section 5.3). “Proposed validation/criterion for upgrade” lists the experiment(s) required to raise a claim to a higher tier. Numbers refer to references in the reference list. AKT, protein kinase B; APC, antigen-presenting cell; ECM, extracellular matrix; EV, extracellular vesicle; MMP, matrix metalloproteinase; MVB, multivesicular body; OSS, oscillatory shear stress; VSMC, vascular smooth muscle cell.

**TABLE 2:** Evidence-grading scheme applied to all key mechanistic claims in this review.

Tier	Designation	Criteria
[E]	Established mechanism	Supported by multiple independent human studies and confirmed by *in vivo* genetic loss-/gain-of-function evidence
[P]	Strong preclinical evidence	Robust murine or large-animal *in vivo* data incorporating mechanistic intervention
[I]	Plausible inference	Extrapolation from *in vitro* mechanistic studies or correlational human data without causal *in vivo* validation
[S]	Speculative extrapolation	Forward-looking hypotheses advanced by this review that await empirical testing

The scheme is applied throughout the text using the bracketed notation [E], [P], [I], and [S], and is used to stratify the claims listed in Table 1.

## 2 Spatial architecture of atherosclerotic plaques

### 2.1 Macroscopic anatomical compartments of atherosclerotic plaques

Mature atherosclerotic plaques are heterogeneous lesions. Pathological examination divides them, at the macroscopic level, into four principal compartments: the fibrous cap, the lipid-rich necrotic core (LRNC), the shoulder region, and the neovascularization zone. Each compartment contributes to overall lesion stability in a different way (Libby, 2021).

The fibrous cap lies between the plaque interior and the arterial lumen. It is built mainly from type I and type III collagen, proteoglycans, and other extracellular matrix (ECM) components secreted by resident smooth muscle cells (SMCs). In an inflammatory environment, activated macrophages release matrix metalloproteinases (MMPs) that degrade these ECM constituents. The cap thins progressively as a result (Newby, 2016).

TCFA is defined by three pathological criteria (Virmani et al., 2006). The fibrous cap must be ≤65 μm at its thinnest point. The underlying necrotic core must occupy a substantial cross-sectional area of the plaque. Dense CD68^+^ macrophage infiltration of the cap must be present. These criteria identify a rupture-prone morphology, but they do not predict imminent rupture in any individual lesion. In autopsy series, most TCFAs heal or stabilise without ever producing a clinical event.

The lipid-rich necrotic core accumulates lipids, necrotic cell debris, free cholesterol crystals, and apoptotic foam cells. It lacks viable cellular scaffolding. Two main mechanisms drive its formation: foam-cell apoptosis followed by secondary necrosis, and impaired efferocytosis that leaves apoptotic cells uncleared (Doran et al., 2020). Cholesterol crystals within the core can directly activate the NLRP3 inflammasome, which amplifies local inflammation and drives further core expansion (Duewell et al., 2010). As the necrotic core enlarges, mechanical stress concentrates beneath the fibrous cap. This is the principal pathological substrate for the transition to a vulnerable phenotype (Ohayon et al., 2014).

The shoulder region sits at the junction between the fibrous cap and the adjacent arterial wall. It bears the highest mechanical stress within the plaque and shows the densest inflammatory cell infiltration. It is therefore the most common rupture site (Tomaniak et al., 2020). Local proteases and pro-inflammatory cytokines act together to thin the cap at this site. An elevated total cholesterol/HDL ratio is an independent risk factor for rupture of lipid-rich plaques (Virmani et al., 1998).

The neovascularization zone is found mainly at the plaque base and shoulders. It comprises thin-walled, structurally immature capillaries derived from proliferating endothelial cells. These vessels are intrinsically leaky, and the resulting erythrocyte extravasation feeds intraplaque iron deposition and oxidative stress (Parma et al., 2017). When fragile neovessels rupture, the intraplaque hemorrhage (IPH) that follows acutely enlarges plaque volume and undermines fibrous cap integrity. IPH is therefore an important trigger of plaque progression and adverse cardiovascular events (Michel et al., 2011).

### 2.2 Spatial cellular distribution within atherosclerotic plaques

Single-cell RNA sequencing (scRNA-seq) and spatial transcriptomics now allow identification and localisation of distinct cellular subpopulations within plaques. This has refined understanding of microenvironmental heterogeneity.

Macrophages are the most abundant and functionally diverse immune cells in plaques, and their subpopulations occupy distinct spatial niches. The conventional M1/M2 binary fails to capture this diversity. Large-scale scRNA-seq meta-analysis has resolved at least seven major macrophage subpopulations within plaques, including the inflammatory NLRP3^+^ subset, the lipid-associated TREM2^+^ subset, the proliferative MKI67^+^ subset, and the tissue-resident FOLR2^+^ subset (Zernecke et al., 2020). TREM2^+^ macrophages are enriched in the peri-necrotic core region. They handle lipid metabolism and express a relatively anti-inflammatory phenotype, and are considered the principal effector cells for clearance within the necrotic core (Jaitin et al., 2019). Single-cell transcriptomics of human carotid plaques has shown that inflammatory macrophage subsets accumulate at the shoulder region whereas TREM2^+^ subsets localise to deeper layers of the necrotic core (Depuydt et al., 2020). This pattern tracks with local inflammatory intensity.

Spatial transcriptomics has clarified the molecular logic of this distribution. Integrative analysis of human coronary plaques indicates that the shoulder region is enriched for activated macrophage signatures (Mosquera et al., 2023). The necrotic-core-adjacent region, by contrast, carries gene modules associated with foam-cell formation, including the lipid uptake and transport genes ABCA1 and CD36. Combining spatial transcriptomic data with genome-wide association findings has confirmed spatial co-localization of TREM2^+^ macrophages with the necrotic core and identified candidate gene loci relevant to plaque structural stability (Slenders et al., 2022).

T lymphocytes within plaques are predominantly CD4^+^ effector T cells and regulatory T cells (Tregs). They are distributed mainly at the shoulder region and in the deeper fibrous cap. Th1-polarized CD4^+^ T cells sustain pro-inflammatory macrophage activation through IFN-γ secretion (Roy et al., 2022). Tregs suppress local inflammatory responses via IL-10 and TGF-β, and their density correlates positively with plaque stability (Saigusa et al., 2020). scRNA-seq has also identified exhausted CD8^+^ T cell subsets within plaques, marked by high PD-1 and TIM-3 expression. This finding points to tumour-like immunosuppressive mechanisms in the plaque microenvironment (Fernandez et al., 2019).

Smooth muscle cells show striking phenotypic plasticity in the plaque setting. Lineage tracing combined with scRNA-seq has described an SMC-derived “fibromyocyte” subpopulation, defined by high LGALS3 and SPP1 expression and located at the plaque shoulder and superficial fibrous cap (Wirka et al., 2019). SMC-to-mesenchymal-like phenotypic switching has been confirmed in both murine and human plaques, with TCF21 identified as the critical transcription factor governing this transition (Pan et al., 2020). The pluripotency-associated genes Klf4 and Oct4 also play central roles in SMC reprogramming during late-stage lesion development (Alencar et al., 2020).

**FIGURE 1:**
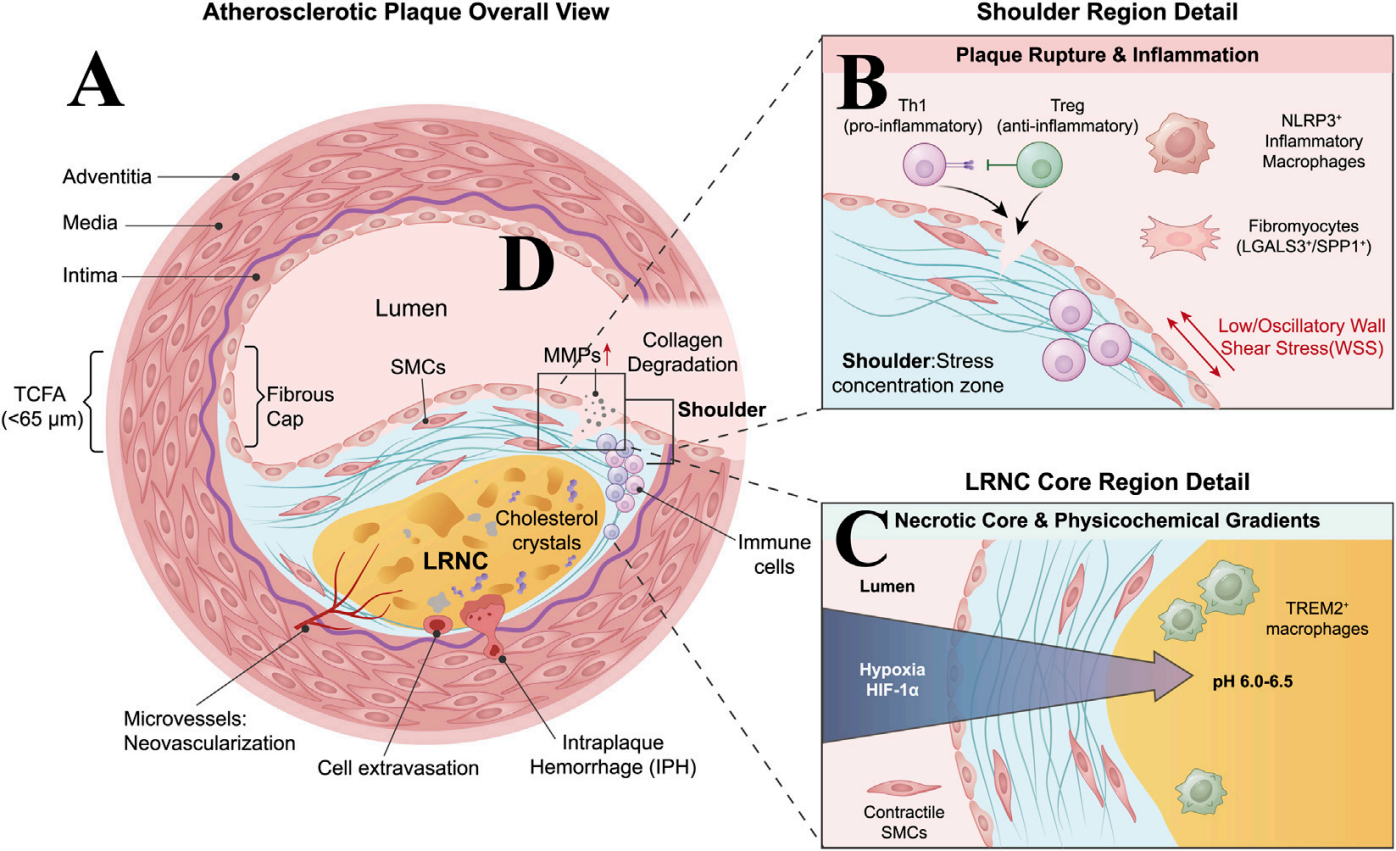
The Spatial Architecture and Microenvironment Heterogeneity of Atherosclerotic Plaques. The schematic illustrates the highly organized spatial complexity of a mature atherosclerotic plaque. **(A)** Macroscopic Anatomical Zones: The plaque is divided into the fibrous cap (SMC-rich protective barrier), the lipid-rich necrotic core (LRNC, containing cholesterol crystals and cellular debris), the shoulder region (the primary site of mechanical stress and rupture), and the neovascularization zone (leaky microvessels associated with intraplaque hemorrhage). **(B)** Cellular Spatial Distribution: Recent spatial transcriptomics and scRNA-seq reveal distinct cell niches. Inflammatory NLRP3+ macrophages and SPP1+ fibromyocytes (SMC-derived) preferentially accumulate in the shoulder region, while TREM2+ lipid-associated macrophages are localized at the LRNC periphery. T cell subsets (Th1 and Tregs) infiltrate the cap and shoulder, modulating local inflammation. **(C)** Physicochemical Gradients: The plaque microenvironment is characterized by low oscillatory wall shear stress (WSS) at the shoulder, a hypoxic gradient (HIF-1α stabilization) and acidic pH (6.0–6.5) toward the deep necrotic core, and regionalized reactive oxygen species (ROS) accumulation. **(D)** Determinants of Plaque Stability: Vulnerable thin-cap fibroatheroma (TCFA) is defined by a cap thickness <65 μm and an LRNC area >40%. The synergistic effect of mechanical stress, extracellular matrix degradation by MMPs, and impaired efferocytosis governs the transition from a stable to a vulnerable phenotype.

Endothelial cells display spatial functional heterogeneity that bears on plaque progression. Luminal endothelial cells exposed to low oscillatory shear stress upregulate the adhesion molecules ICAM-1 and VCAM-1, supporting monocyte transendothelial migration (Gimbrone and García-Cardeña, 2016). Endothelial cells inside the intraplaque neovascularization zone behave very differently from luminal endothelium. Their structural immaturity and inherent leakiness drive intraplaque hemorrhage and inflammatory cell infiltration (Parma et al., 2017). Figure 1 summarises this spatial organisation—macroscale anatomy, cellular distribution, physicochemical gradients, and stability determinants. It also illustrates how mechanical strain, matrix degradation, and impaired efferocytosis converge at the shoulder to favour the transition from stable plaque toward thin-cap fibroatheroma (TCFA).

### 2.3 Physicochemical characteristics of the plaque microenvironment

The plaque interior is physicochemically heterogeneous. The resulting microenvironmental gradients shape cellular function and signal transduction within the lesion.

Mechanical heterogeneity is the most important physical feature. Wall shear stress (WSS) generated by blood flow is unevenly distributed across the plaque surface. The shoulder region typically experiences low oscillatory WSS, whereas the plaque apex and adjacent normal arterial segments are exposed to relatively higher shear stress (Chatzizisis et al., 2007). Low oscillatory WSS activates pro-inflammatory pathways such as NF-κB and AP-1 in endothelial cells, and suppresses the anti-inflammatory transcription factors KLF2 and KLF4. This promotes inflammatory cell recruitment and lesion progression (Givens and Tzima, 2016). Matrix stiffness also varies regionally. The elastic modulus is approximately 5–50 kPa in normal artery wall. It may reach 200–500 kPa in the fibrous cap, and can reach the megapascal range in calcified zones (Holzapfel et al., 2004). Low WSS combined with elevated peak circumferential stress at the shoulder favours necrotic core enlargement, and this biomechanical combination is a key determinant of structural instability (Pedrigi et al., 2016).

Hypoxic gradients are a second key feature. Because the plaque volume is large and the intraplaque vasculature is incomplete, oxygen diffusion to deeper layers is impaired. A pronounced hypoxic microenvironment develops in the necrotic core and surrounding regions, with local oxygen tension potentially falling below 5 mmHg (Marsch et al., 2013). Hypoxia stabilises hypoxia-inducible factor-1α (HIF-1α), which activates downstream programmes including enhanced glycolysis, VEGF-mediated neovascularization, and increased macrophage pro-inflammatory activity. Histological studies of human carotid plaques show that the spatial distribution of HIF-1α target gene expression closely tracks hypoxic regions, with a positive correlation between hypoxia and inflammatory marker abundance (Sluimer et al., 2008).

Local pH gradients arise from active inflammatory metabolism, anaerobic glycolysis, and cholesterol crystal deposition, which together acidify the intraplaque environment. The pH within the necrotic core can fall to 6.0–6.5, well below physiological tissue pH (7.35–7.45). This acidic milieu reduces efferocytosis efficiency, promotes NLRP3 inflammasome activation with IL-1β secretion, and increases lysosomal protease activity. The combined effect deepens local inflammation and lipid accumulation (Grebe et al., 2018).

Oxidative stress is regionally concentrated. Reactive oxygen species (ROS) are produced mainly by activated macrophages via the NADPH oxidase pathway, by dysfunctional endothelial cells, and by uncoupled mitochondrial respiratory chains. ROS reach their highest concentrations in the densely inflamed shoulder region and the peri-necrotic core zone (Griendling et al., 2000). ROS accelerate foam cell formation by oxidising LDL to oxidized LDL (oxLDL). They also impair fibrous cap collagen metabolism by activating redox-sensitive signaling. Both effects compromise plaque structural integrity (Libby et al., 2018).

### 2.4 Relationship between spatial architecture and plaque stability

Spatial structural features of atherosclerotic plaques are important determinants of stability. Clinical-pathological research has produced several morphological parameters now used for vulnerability assessment.

Fibrous cap thickness remains the most clinically validated single morphological predictor of rupture risk. The pathological diagnosis of TCFA—a fibrous cap ≤65 μm overlying a necrotic core with abundant inflammatory infiltrate—derives from the Virmani criteria (Virmani et al., 2006). Autopsy work has documented a strong pathological association between TCFA and acute coronary thrombotic events, and has identified an elevated total cholesterol/HDL ratio as an independent risk factor for rupture of lipid-rich plaques (Virmani et al., 1998). Prospective optical coherence tomography (OCT) studies have further shown that OCT-detected TCFA independently predicts adverse clinical events in diabetic patients with non-ischemic lesions. TCFA-positive patients show markedly higher rates of major adverse cardiovascular events (Kedhi et al., 2021).

Necrotic core size and plaque instability are consistently linked across pathological and imaging studies. Enlargement of the necrotic core raises stress concentration beneath the fibrous cap and reduces the overall mechanical resilience of the lesion (Ohayon et al., 2014). Cholesterol crystals within the core have two effects. They activate the NLRP3 inflammasome and initiate local inflammatory cascades. They also inflict mechanical microtrauma on the fibrous cap under loading, which accelerates rupture (Duewell et al., 2010).

The spatial density of inflammatory cells is a further important parameter. Pathological studies show an inverse correlation between intraplaque CD68^+^ macrophage density and fibrous cap thickness, consistent with local inflammatory infiltration driving progressive cap thinning (Naghavi et al., 2003). Shoulder region macrophage density has been incorporated into several plaque vulnerability scoring systems. High-density infiltration is closely associated with elevated local MMP secretion, accelerated collagen degradation, and increased rupture risk (Johnson, 2017). Recent spatial transcriptomic work has shown that multiple PDGFRβ-expressing cell types cooperate, through coordinated bioenergetic mechanisms, to build and remodel the atherosclerotic fibrous cap. Such multi-cellular cooperation within the plaque microenvironment may yield therapeutic targets for plaque-stabilising strategies (Newman et al., 2021).

In summary, macroscopic anatomical compartmentalization, the spatial cellular distribution map, and the physicochemical features of the plaque microenvironment are tightly interrelated. Together they form the regulatory network that governs plaque stability. Spatial heterogeneity should be read as a necessary but not sufficient condition for plaque rupture. Many plaques with disorganised spatial architecture remain clinically silent for decades, indicating that additional triggering factors—acute hemodynamic stress or systemic inflammatory flares—are required for an event to occur. A clearer understanding of how spatial architecture relates to stability supports current TCFA imaging diagnostic criteria. It also provides a theoretical basis for microenvironment-targeted plaque-stabilisation strategies. The spatial architecture described in this section is more than a descriptive backdrop. It constitutes the structural template upon which the two regulatory axes treated in Sections 3, 4 — mechanotransduction and vesicular trafficking—are imposed. Sections 5, 6 will draw these strands together as the Mechano-Vesicular Dual-Axis (MVDA) framework, in which the loss of coherent coupling between the two axes, rather than any single morphological defect, marks the transition from stable to vulnerable plaque.

## 3 Mechanotransduction in plaque immune regulation

### 3.1 The mechanical microenvironment within atherosclerotic plaques

Atherosclerotic plaques develop preferentially at arterial bifurcations and curved segments, where the local mechanical microenvironment differs sharply from straight, undisturbed arterial regions. At such sites, oscillatory shear stress (OSS) — typically below 4 dyne/cm^2^ — is the cardinal biomechanical feature driving atherogenesis, whereas laminar shear stress (LSS, 10–20 dyne/cm^2^) in straight arterial segments is atheroprotective (Zhou et al., 2023). The two flow patterns engage distinct intracellular signaling cascades, directing endothelial cells toward pro- or anti-inflammatory phenotypes and so shaping plaque initiation and progression.

Matrix stiffness within plaques also evolves dynamically with disease progression. The stiffness gradient already detailed in Section 2.3 — from ∼5 to 50 kPa in normal artery wall up to the megapascal range in calcified zones (Holzapfel et al., 2004) — is superimposed upon this hemodynamic landscape (Kwak et al., 2014), so that cells in different plaque regions are exposed to qualitatively different mechanical loads and respond in a cell-type-specific manner.

#### 3.1.1 Hierarchy of mechanotransduction axes

Among the numerous signaling cascades engaged by mechanical stimuli, three constitute the dominant regulatory axes in plaque immunology: (i) Piezo1–Ca^2+^–CaMKII as the primary mechano-electrical sensor; (ii) Integrin–FAK–RhoA/ROCK as the cytoskeletal mechanotransducer; and (iii) Hippo–YAP/TAZ as the mechano-transcriptional output node. The PI3K/Akt/mTOR cascade and the KLF2/4 transcriptional programme function as integrative modulators that gate the output of these dominant axes. All other pathways referenced in this section operate as context-dependent fine-tuning modules. This three-tier hierarchy is used throughout Sections 3.2–3.5 as the organising frame: each cellular response is traced first to one of the three dominant axes, with modulators and fine-tuning modules then layered on.

### 3.2 Mechanosensing and inflammatory activation in endothelial cells

#### 3.2.1 Piezo1-mediated pro-inflammatory signaling under OSS

Piezo1 is a mechanosensitive cation channel expressed on the endothelial cell membrane, with elevated expression in atherosclerotic lesions and at sites of disturbed blood flow. OSS-induced Piezo1 activation triggers Ca^2+^ influx and engages the Ca^2+^/CaM/CaMKII cascade; the signal is then relayed through FAK/Src kinases to Yes-associated protein (YAP), upregulating the adhesion molecules VCAM-1 and ICAM-1 and amplifying endothelial inflammation (Lan et al., 2024). Pharmacological inhibition of Piezo1 with the peptide GsMTx4 attenuates endothelial inflammation and delays plaque progression in ApoE^−/−^ mice (Lan et al., 2024) [P].

#### 3.2.2 Atheroprotective signaling via the Piezo1–CaMKII–MEKK3–ERK5–KLF2/4 axis

Under LSS, Piezo1 mediates atheroprotective signaling. LSS-activated Piezo1 triggers Ca^2+^ influx and CaMKII autophosphorylation; CaMKII then interacts with MEKK3 to activate the MEK5/ERK5 cascade, which induces the transcription factors KLF2 and KLF4 (Zheng et al., 2022). KLF2/4 are central regulators of endothelial anti-inflammatory homeostasis and suppress NF-κB signaling and its downstream targets. Endothelial-specific Piezo1 knockout mice show markedly reduced KLF2/4 expression in the thoracic aorta, identifying the Piezo1–CaMKII–MEKK3–ERK5 axis as the molecular link between shear stress sensing and KLF2/4 induction (Zheng et al., 2022) [E].

#### 3.2.3 Negative regulation of KLF2/4 by γ-protocadherins

γ-Protocadherins are upregulated in the endothelium of human atherosclerotic lesions. After proteolytic cleavage of the intracellular domain (ICD) and its translocation to the nucleus, the γ-protocadherin ICD associates with the Notch intracellular domain (NICD) and suppresses its transcriptional activity, blunting the KLF2/4-driven endothelial anti-inflammatory programme (Joshi et al., 2024). In murine atherosclerosis models, genetic deletion and antibody blockade of γ-protocadherins reduce plaque burden without compromising host defence (Joshi et al., 2024). These findings point to a pro-inflammatory amplification mechanism upstream of KLF2/4 with potential therapeutic relevance [P].

#### 3.2.4 Mechano-metabolic coupling: The KLF4–GCKR–AMPK axis

LSS regulates not only inflammatory signaling but also endothelial glycolysis, through a KLF4–GCKR–AMPK axis that couples mechanics to metabolism. Pulsatile laminar flow induces KLF4 to act as a pioneer transcription factor, binding the glucokinase regulatory protein (GCKR) promoter to remodel chromatin and upregulate GCKR; flow-activated AMPK simultaneously phosphorylates GCKR at Ser-481, strengthening its interaction with hexokinase HK1 and so inhibiting glycolytic flux, helping to keep the endothelium oxidatively poised (Han et al., 2021). OSS, in contrast, activates HIF-1α to promote aerobic glycolysis, producing a pro-inflammatory metabolic state with the opposite polarity to that maintained by LSS.

### 3.3 Macrophage mechanosensing and polarization

#### 3.3.1 Matrix stiffness regulates macrophage polarization via the Piezo1–YAP axis

Matrix stiffness is an important physical determinant of macrophage polarization. On soft to medium-stiffness substrates, macrophages adopt an anti-inflammatory M2-like phenotype, whereas on stiff substrates Piezo1 expression rises, YAP translocates to the nucleus, and polarization shifts toward a pro-inflammatory M1-like state (Wang Y. et al., 2022; Mei et al., 2024). Mechanistic work has shown that matrix stiffening drives this shift through the Piezo1–YAP signaling axis, and the YAP–TEAD inhibitor verteporfin can return macrophages to the M2 state on stiff substrates (Mei et al., 2024). Within the plaque, the heterogeneous local stiffness distribution shapes the spatial pattern of macrophage polarization: softer zones (e.g., the peri-necrotic core) tend to favour M2 maintenance, while stiffer fibrotic and calcified zones favour M1 polarization, contributing to the regional immune topology of the lesion [P].

#### 3.3.2 Mechanical microenvironment-driven metabolic reprogramming of macrophages

Mechanical cues also reshape macrophage function through cell metabolism. Pro-inflammatory macrophages depend largely on aerobic glycolysis, while anti-inflammatory macrophages favour oxidative phosphorylation, and regional differences in the plaque mechanical microenvironment can set these metabolic and inflammatory outputs through YAP/TAZ and downstream metabolic networks (Wang J. et al., 2024).

### 3.4 Mechanosensitive phenotypic switching in vascular smooth muscle cells

#### 3.4.1 Physiological cyclic strain and maintenance of the contractile phenotype

Under physiological conditions, cyclic strain (5%–10% elongation, 1 Hz) activates the SRF–myocardin transcriptional axis and keeps vascular smooth muscle cells (VSMCs) in a contractile state. Pathological high-magnitude strain activates the RhoA/ROCK cascade, downregulates contractile genes (SM-MHC, smooth muscle actin) while upregulating KLF4, and drives VSMCs toward a synthetic phenotype with greater intimal migration and ECM secretion, aggravating plaque remodelling (Sawma et al., 2022).

#### 3.4.2 ECM stiffness regulates phenotypic selection via Rac–RhoA balance

ECM stiffness governs VSMC phenotypic fate through the Rac–RhoA balance. Stiffer environments promote RhoA activation, supporting actin polymerization and MKL nuclear translocation that reinforce contractile gene transcription, whereas Rac activation favours proliferation. As atherosclerosis progresses, sustained matrix stiffening and VSMC dedifferentiation track one another closely, and each tends to sustain the other (Elmarasi et al., 2024) [P].

#### 3.4.3 Osteogenic transdifferentiation and the calcification feedback

Dedifferentiated synthetic-phenotype VSMCs can undergo further osteogenic transdifferentiation. The osteogenic transcription factor RUNX2, acting together with WNT/β-catenin and BMP signaling, drives hydroxyapatite deposition and plaque calcification (Sutton et al., 2023). The calcification that ensues raises local matrix stiffness still further, and the increased stiffness tends to keep VSMCs in the osteogenic state—a closed-loop relationship in which calcification and stiffness sustain one another, with the practical consequence that established calcified regions resist reversal.

#### 3.4.4 Dual-pathway integration of pressure and stiffness sensing

VSMCs sense hydrostatic pressure and matrix stiffness in parallel, and the two mechanical signals coordinate cytoskeletal dynamics through distinct, partly overlapping pathways. Stiffness signals act mainly through a RhoA–ROCK2–LIMK2–cofilin axis to regulate actin severing, whereas hydrostatic pressure signals lower cofilin phosphorylation by independent routes; the integration of the two pathways determines cytoskeletal tension and ultimately phenotypic fate (Swiatlowska et al., 2022).

### 3.5 Mechanotransduction and spatially directed immune cell migration

#### 3.5.1 Macrophage durotaxis

Directed cell migration along stiffness gradients toward stiffer substrates is termed durotaxis. The process depends on asymmetric integrin–focal adhesion distribution and polarized Rac GTPase activation; on stiffness-graded substrates, macrophages migrate preferentially toward stiffer zones such as the fibrous cap, providing a mechanism by which pro-inflammatory cells accumulate in vulnerable regions of the plaque (Shellard and Mayor, 2021) [I].

#### 3.5.2 Mechano-chemical migration synergy at the plaque shoulder

The plaque shoulder combines low OSS with high ECM stiffness; OSS-activated endothelial cells upregulate ICAM-1 and VCAM-1, supporting monocyte adhesion and transendothelial migration (He et al., 2022). Together, the mechanical and chemical cues at this site form a mechano-chemical coupling that favours pro-inflammatory immune cell accumulation in the most vulnerable plaque regions.

#### 3.5.3 Substrate-dependent durotaxis of VSMCs

VSMC durotaxis depends strictly on ECM composition: fibronectin-coated stiffness-gradient substrates support directed VSMC migration toward stiffer regions, whereas laminin-coated substrates do not (Hartman et al., 2016). Altered ECM composition and elevated stiffness therefore act together to drive directed VSMC aggregation and intimal infiltration in atherosclerotic lesions.

#### 3.5.4 Role of Piezo1 in transendothelial migration

Piezo1 senses compressive cell deformation and tunes Ca^2+^-dependent cytoskeletal reorganisation to facilitate monocyte transmigration across the endothelial barrier. OSS-induced Piezo1 activation in endothelial cells raises endothelial permeability and couples directly to selective transendothelial immune cell infiltration, further amplifying local plaque inflammation (Lan et al., 2024) [I].

**FIGURE 2:**
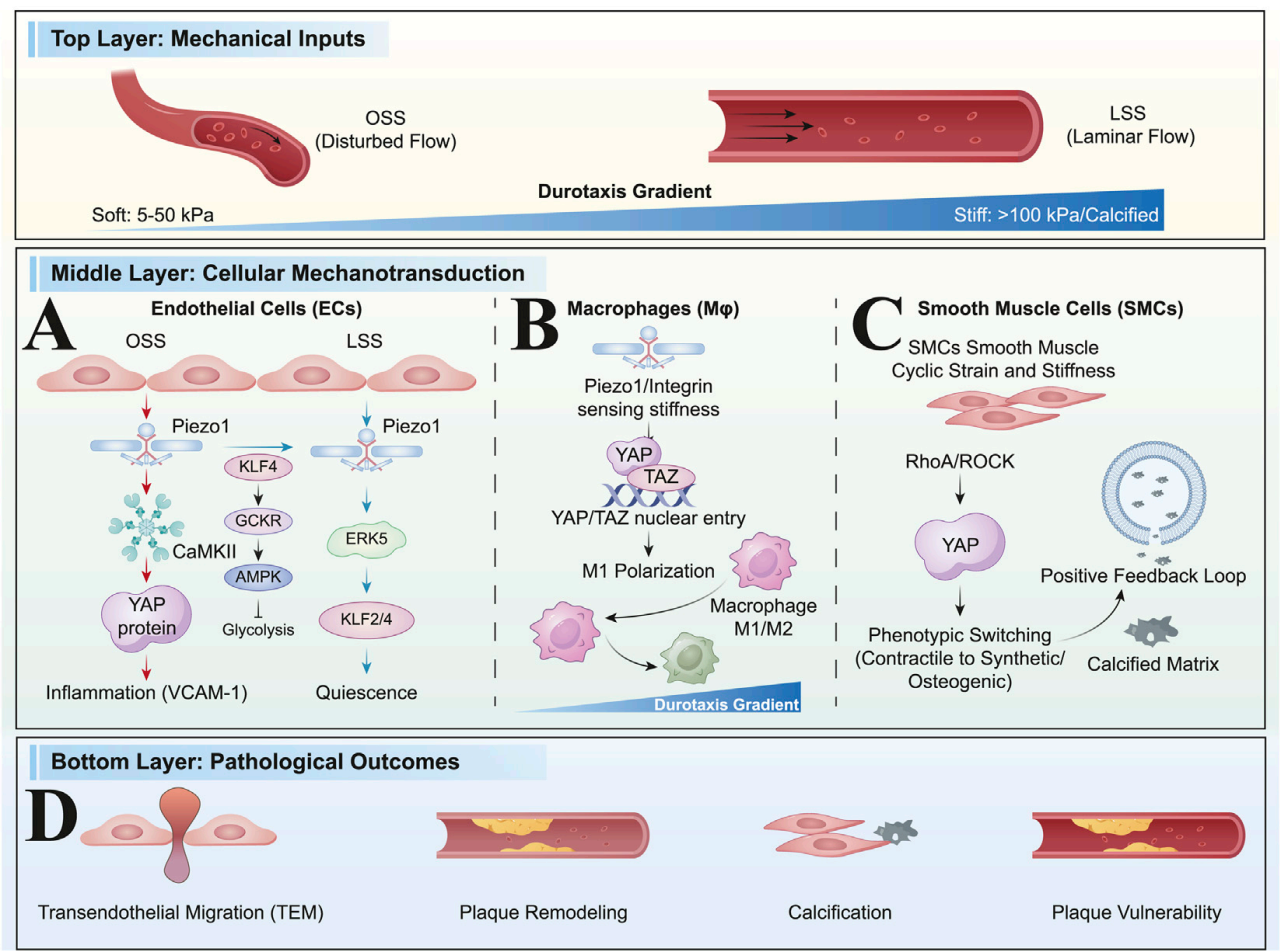
Mechanotransduction Mechanisms Regulating Immune Responses and Cell Phenotypes in Atherosclerosis. The figure integrates the multi-axial mechanical regulation of vascular cells within the plaque microenvironment. **(A)** Endothelial Mechanosensing: Oscillatory shear stress (OSS) activates the Piezo1-CaMKII-YAP axis to promote inflammation and aerobic glycolysis (HIF-1α dependent). In contrast, laminar shear stress (LSS) triggers the Piezo1-ERK5-KLF2/4 pathway and the KLF4-GCKR-AMPK axis, maintaining endothelial quiescence and oxidative metabolism. γ-Protocadherin acts as a negative regulator of KLF2/4. **(B)** Macrophage Polarization and Durotaxis: Increasing matrix stiffness activates Piezo1 and the Hippo-YAP/TAZ pathway, driving macrophages toward a pro-inflammatory M1-like phenotype and metabolic reprogramming. Macrophages also exhibit durotaxis, migrating toward stiffer regions (e.g., the fibrous cap) via integrin-mediated focal adhesions. **(C)** VSMC Phenotypic Switching: Pathological strain and high stiffness activate RhoA/ROCK and YAP/TAZ signaling, inducing a switch from a contractile to a synthetic or osteogenic phenotype (Runx2+). This process promotes extracellular matrix (ECM) remodeling and calcification, the latter of which creates a positive feedback loop by further increasing local stiffness. **(D)** Integrated Effects: The synergy between mechanical forces (OSS, stiffness) and chemical signals (adhesion molecules) facilitates monocyte transendothelial migration and local immune cell accumulation, ultimately determining plaque stability and the risk of rupture.

Figure 2 summarises the mechanotransduction mechanisms governing vascular cell behaviour within the atherosclerotic plaque microenvironment. Panel A shows how OSS activates the Piezo1–CaMKII–YAP axis to drive endothelial inflammation and HIF-1α-dependent glycolysis, while LSS engages the Piezo1–ERK5–KLF2/4 and KLF4–GCKR–AMPK pathways to maintain endothelial quiescence. Panel B depicts how matrix stiffness activates Piezo1 and Hippo–YAP/TAZ to polarise macrophages toward a pro-inflammatory M1-like phenotype and direct their durotactic migration through integrin-mediated focal adhesions. Panel C illustrates how pathological strain and elevated stiffness engage RhoA/ROCK and YAP/TAZ in VSMCs, driving a phenotypic switch toward synthetic or osteogenic (Runx2^+^) states and promoting calcification, which in turn raises local stiffness. Panel D integrates these responses, showing how mechanical and chemical signals act together to facilitate monocyte transendothelial migration and to set plaque vulnerability and rupture risk.

## 4 The role of vesicular trafficking in plaque immune communication

### 4.1 Types and origins of vesicles in the plaque microenvironment

Extracellular vesicles (EVs) are nanometer-to-micrometer-scale particles released by cells and enclosed by a phospholipid bilayer. The MISEV2023 guidelines of the International Society for Extracellular Vesicles define EVs as lipid-bilayer-bounded particles that cannot replicate autonomously, and recommend operational terminology—small EVs (sEVs) and large EVs (lEVs) — over the legacy descriptors “exosomes,” “microvesicles,” and “apoptotic bodies,” whose biogenetic origins often cannot be reliably assigned from the experimental data alone (Welsh et al., 2024). Because the legacy terms remain widely used in primary studies, this review retains them only where the cited primary study provides explicit biogenesis evidence, and uses sEVs/lEVs otherwise.

#### 4.1.1 Terminology convention

Following MISEV2023 (Welsh et al., 2024), throughout this review we use the operational terms small EVs (sEVs, <200 nm) and large EVs (lEVs, >200 nm) as default descriptors. The legacy terms “exosomes,” “microvesicles,” and “apoptotic bodies” are retained *only* when the cited primary study provided explicit biogenesis evidence—for example, live MVB-fusion imaging for exosomes, or caspase-dependent membrane blebbing for apoptotic bodies. When the original work characterised EVs solely by size or surface markers without biogenesis tracing, we substitute the operational term and annotate the cited finding accordingly. Readers should therefore interpret “exosome” and “microvesicle” in this review as biogenesis-confirmed subsets of sEVs and lEVs, not as default descriptors of any small or large particle.

Within the biogenesis-defined framework: exosomes (30–150 nm) originate from intracellular multivesicular bodies (MVBs) via the endosomal pathway, are released upon MVB–plasma-membrane fusion, and are enriched in tetraspanins CD9, CD63, CD81 and Hsp70 (Welsh et al., 2024). Microvesicles (100–1,000 nm) are generated by direct budding of the plasma membrane; their secretion depends on elevated cytosolic Ca^2+^ and on suppression of phospholipid flippase activity, which externalises phosphatidylserine (PS) on the outer leaflet (Olejarz et al., 2023). Apoptotic bodies, the largest subtype (500 nm–5 μm), form during apoptosis through cytoskeletal contraction and membrane blebbing; their surfaces carry “eat-me” signals such as PS and calreticulin, and under physiological conditions they are efficiently cleared by phagocytes (Shu et al., 2024; Wang Z. et al., 2022).

In the plaque microenvironment, multiple cell types release EVs whose quantity and cargo composition depend on cell activation state and local stimuli.

Endothelial cells (ECs) markedly increase EV secretion in response to ox-LDL, oscillatory shear stress, and TNF-α, and the cargo shifts from anti-inflammatory toward pro-inflammatory, supporting downstream monocyte activation, endothelial dysfunction, and plaque destabilisation (He et al., 2018). EVs released by endothelial cells under disturbed flow deliver pro-inflammatory cargo to macrophages through the MAPK pathway, driving M1 polarisation and accelerating atherosclerosis (Hou et al., 2025).

Macrophages are the principal producers of EVs within plaques. Foam-cell macrophages stimulated by ox-LDL or hypoxia release abundant sEVs enriched in pro-inflammatory miRNAs and oxidised lipids; these EVs transmit pro-atherogenic signals to neighbouring cells and act in a retrograde fashion on endothelial cells, supporting further monocyte recruitment (Olejarz et al., 2023).

Vascular smooth muscle cells (VSMCs) increase EV secretion after phenotypic switching to the synthetic state. Under pro-atherogenic conditions, VSMC-derived EVs carry calcification-associated proteins including annexin A6 and alkaline phosphatase, serving as key nucleation sites for hydroxyapatite deposition within plaques (Kapustin et al., 2015; Yang et al., 2019).

Platelets are the most abundant source of EVs in the circulation. Platelet-derived EVs (pEVs) are released in large quantities on thrombin activation and express P-selectin, activated glycoprotein IIb/IIIa, and PS, with combined pro-coagulant and pro-inflammatory activities; pEVs can penetrate atherosclerotic plaques and interact with multiple intraplaque cell types (Weiss et al., 2025; Ma et al., 2021).

EV secretion within plaques is temporally and spatially regulated. EV subtypes and the directionality of their cargo respond dynamically to local oxygen tension, shear stress, lipid load, and the cytokine network, making EVs effective mediators of plaque immune communication (Welsh et al., 2024; Olejarz et al., 2023).

#### 4.1.2 Methodological standards for plaque-EV studies

Reliable interpretation of plaque-derived EV data requires adherence to MISEV2023 reporting standards (Welsh et al., 2024): (i) separation by combined size-exclusion chromatography and density gradient ultracentrifugation, rather than single-step ultracentrifugation alone, which co-isolates lipoproteins abundant in atherosclerotic plasma; (ii) marker characterisation including ≥3 EV-positive markers (e.g., CD9, CD63, CD81, syntenin-1) and ≥1 negative marker (e.g., calnexin) to exclude cellular contamination, with apoB and apoA-I quantified to assess lipoprotein contamination; (iii) quantification through complementary techniques (NTA in combination with single-EV imaging, e.g., ExoView) rather than NTA alone; (iv) normalisation to particle number, total protein, and parental-cell number rather than volume alone, given the marked variation in plasma lipoprotein burden across atherosclerotic patient cohorts; and (v) reporting through EV-TRACK to support cross-study comparability. Compliance with these standards is essential before any EV-derived biomarker can advance toward clinical translation.

### 4.2 Vesicle cargo composition and functional information transfer

The functional molecules carried by EVs—proteins, miRNAs, lncRNAs, lipids, and metabolites—reflect the activation state of the parent cell and, on internalisation, can directly reprogramme gene expression and the functional phenotype of recipient cells (Buzas, 2023; Davidson et al., 2023).

#### 4.2.1 Pro-inflammatory miRNAs

miR-155 is among the most intensively studied pro-inflammatory miRNAs in plaque-derived EVs. EVs from ox-LDL-treated endothelial cells are enriched in miR-155 and deliver it to monocytes and macrophages, supporting a shift from the M2 anti-inflammatory phenotype toward an M1 pro-inflammatory state and amplifying intimal inflammation. EVs from KLF2-activated endothelial cells, by contrast, contain less miR-155 and exert the opposite effect (He et al., 2018). Neutrophil microvesicles accumulate at sites of disturbed flow, are internalised by endothelial cells, and the miR-155 they carry activates NF-κB signaling, promotes inflammatory gene expression, and accelerates leukocyte adhesion and plaque progression (Gomez et al., 2020). Exosomes from nicotine-stimulated macrophages are enriched in miR-21-3p, which targets PTEN, activates PI3K/AKT, and promotes VSMC migration and proliferation, accelerating neointima formation (Zhu et al., 2019) [P].

#### 4.2.2 Anti-inflammatory miRNAs

miR-146a suppresses NF-κB signaling by targeting TRAF6 and IRAK1; within atherosclerotic plaques it limits foam cell formation and pro-inflammatory cytokine release, with stabilising effects (Chu et al., 2022). EVs derived from endothelial cells under laminar shear stress (LSS-EVs) are enriched in miR-34c-5p, which targets the TGF-β/Smad3 axis and reprogrammes M1 macrophages toward an M2 phenotype (Li et al., 2025).

#### 4.2.3 Oxidised lipids and bioactive phospholipids

Plaque-derived EVs—particularly those released by foam cells and apoptotic cells—are enriched in oxidised phospholipids (oxPL) and lysophosphatidylcholine (LysoPC), which activate TLR signaling and support inflammatory responses in endothelial cells and macrophages (Shu et al., 2024; Zhu et al., 2019). pEVs are enriched in phosphatidylserine and lysophosphatidic acid (LPA), giving them pro-thrombotic activity and engaging endothelial inflammatory signaling (Weiss et al., 2025; Ma et al., 2021).

#### 4.2.4 Heat shock proteins and MHC class II molecules

Heat shock proteins Hsp60 and Hsp70 are ubiquitously present in plaque-derived EVs and act as endogenous damage-associated molecular patterns (DAMPs), engaging TLR2/TLR4 and inducing macrophage production of IL-1β and TNF-α (Davidson et al., 2023). MHC class II molecules expressed on exosomal membranes can present antigen directly to CD4^+^ T cells, supporting adaptive immune activation within plaques without the need for conventional antigen-presenting cell intermediaries (Buzas, 2023) [I].

Overall, EV cargo composition tracks the activation state of the parent cell: EVs from pro-inflammatory cells carry pro-inflammatory miRNAs and oxidised lipids and tend to favour disease progression, whereas EVs from atheroprotected cells carry anti-inflammatory miRNAs and tend toward plaque stabilisation (Buzas, 2023; Davidson et al., 2023).

### 4.3 EV-mediated intercellular immune communication

#### 4.3.1 Endothelial cells → monocytes/macrophages

Activated endothelial cells release EVs carrying pro-inflammatory lncRNAs and miRNAs (including miR-155), which are preferentially taken up by monocytes. EVs from ox-LDL-stimulated ECs, enriched in miR-155, drive the phenotypic conversion of monocytes and macrophages from M2 to M1, intensifying local inflammatory signaling (He et al., 2018). EVs released by endothelial cells under disturbed flow deliver pro-inflammatory cargo to macrophages through the MAPK pathway, driving M1 polarisation and accelerating atherosclerosis (Hou et al., 2025), whereas LSS-EVs enriched in miR-34c-5p promote M2 polarisation (Li et al., 2025). Monocyte- and macrophage-derived EVs can also act on endothelial cells in the reverse direction, upregulating ICAM-1, VCAM-1, IL-6, and IL-8, supporting an inflammatory amplification loop that recruits additional monocytes into the plaque (Olejarz et al., 2023).

#### 4.3.2 Macrophages → T Cells

Macrophage-derived EVs (MDEVs) express surface MHC class II molecules and can present ox-LDL-derived lipid antigens or heat shock protein peptides directly to intimal CD4^+^ T cells, activating adaptive immune responses without classical antigen-presenting cell intermediaries (Buzas, 2023). Exosomes from M1 macrophages are enriched in miR-155, which after uptake by CD4^+^ T cells promotes IFN-γ upregulation and drives Th1 differentiation. M2 macrophage-derived exosomes (BMDM-IL-4-exo) carry miR-99a, miR-146b, and miR-378a, target NF-κB and TNF-α signaling to induce M2 polarisation in recipient macrophages, and regulate hematopoiesis to reduce circulating pro-inflammatory myeloid cells, attenuating atherosclerosis at the systemic level (Bouchareychas et al., 2020). ExoM2 also suppresses PDGF-BB-induced VSMC proliferation and migration, preventing the contractile-to-synthetic transition and reducing plaque burden in ApoE^−/−^ mice (Wang S. et al., 2024) [P].

#### 4.3.3 SMCs ↔ macrophages

On stimulation by calcium overload, inflammatory cytokines, or pro-atherogenic signals, VSMCs undergo osteogenic transdifferentiation and release EVs enriched in alkaline phosphatase (ALP), osteopontin (OPN), and annexin A6 — vesicles that serve as nucleation sites for hydroxyapatite deposition within the plaque (Kapustin et al., 2015; Yang et al., 2019). VSMC-derived EVs are enriched in the PS/annexin A6 complex but lose normal calcification inhibitors such as matrix Gla protein (MGP), so that they behave as pro-mineralising vesicles. On internalisation by macrophages, this calcification-associated cargo triggers NLRP3 inflammasome activation, IL-1β secretion, and aggravation of chronic plaque inflammation (Olejarz et al., 2023; Yang et al., 2019). Exosomes from nicotine-stimulated macrophages, in turn, carry miR-21-3p, which activates PI3K/AKT by targeting PTEN and promotes VSMC migration and proliferation, accelerating neointima formation (Zhu et al., 2019). EV-mediated cross-talk in both directions thus couples plaque calcification and chronic inflammation in a way that is difficult to interrupt by targeting either pathway alone.

### 4.4 Spatial selectivity of vesicular trafficking

#### 4.4.1 Governing principle

We posit that vesicular communication within plaques is *not stochastic* but spatially encoded by a hierarchy of determinants. In decreasing order of dominance: (1) local hemodynamics—OSS-dependent EV residence time and adhesion; (2) matrix density and stiffness gradients—size-selective diffusion barriers; (3) receptor–ligand specificity on recipient cells—PS/MerTK, CD36, integrins; and (4) cytokine and chemokine micro-gradients—modulators of uptake kinetics. Mechanisms (1)–(2) act as dominant drivers that establish the spatial template, while (3)–(4) function as modulators that fine-tune the local information flux. Read in this hierarchy, the subsections below are not a catalogue of independent factors but successive refinements of a single, mechanically-anchored spatial code.

The diffusion, capture, and internalisation of EVs within plaques are regulated by local hemodynamics, matrix density, receptor expression profiles, and cytokine gradients, together forming a spatially addressed directional communication system (Buzas, 2023; Deng et al., 2019).

Oscillatory shear stress (OSS) governs both the volume of EV secretion from endothelial cells and the residence time and directional adhesion of circulating EVs in the vascular lumen. Integrins, P-selectin ligands, and PS on the EV surface bind cognate receptors on injured or activated endothelial cells, so that EVs preferentially accumulate at the low-shear shoulder and curvature regions of the plaque, amplifying local inflammatory signals (Buzas, 2023; Deng et al., 2019).

The dense fibrotic extracellular matrix within plaques—built mainly of type I/III collagen and proteoglycans—acts as a spatial barrier that restricts diffusion of large EVs, while smaller exosomes penetrate the matrix more effectively to reach deep-lying cells (Deng et al., 2019). Increased matrix stiffness lowers the diffusion coefficient of EVs and supports their accumulation in stiffer, denser regions; combined with macrophage durotaxis along stiffness gradients (Section 3.5.1), this concentrates pro-inflammatory cells in vulnerable plaque zones.

PS-rich EVs are preferentially internalised by macrophages that highly express PS recognition receptors such as MerTK, Tim4, and Axl (Shu et al., 2024; Wang Z. et al., 2022). Under pro-inflammatory conditions, macrophages upregulate the scavenger receptors CD36 and SR-A and so take up oxidised-lipid-rich EVs more readily, linking foam cell formation and inflammatory activation (Olejarz et al., 2023; Buzas, 2023).

pEVs, by virtue of expressing platelet membrane proteins including glycoprotein IIb/IIIa and P-selectin, can penetrate atherosclerotic plaques and target sites of fibrin and collagen deposition; this gives them a spatial targeting advantage that has been explored for pEV-based nanoplatforms in plaque-targeted drug delivery (Weiss et al., 2025; Ma et al., 2021) [I].

### 4.5 Impaired apoptotic body clearance and secondary necrosis

Under physiological conditions, apoptotic cells are efficiently cleared by professional phagocytes through efferocytosis, which depends on the precise coordination of multiple molecular recognition systems: the PS–MerTK/Tim4/Axl axis, the calreticulin–LRP1 axis, and the MFG-E8-bridged PS–αvβ3 integrin system (Shu et al., 2024; Wang Z. et al., 2022; Thorp et al., 2008). Effective efferocytosis triggers phagocytes to secrete anti-inflammatory mediators such as TGF-β and IL-10, supporting the resolution of inflammation (Shu et al., 2024; Li et al., 2024).

During atherosclerotic plaque progression, this clearance machinery undergoes systemic impairment, with apoptotic cell accumulation and secondary necrosis as a consequence—a central pathological mechanism underlying plaque instability (Yurdagul et al., 2017; Li et al., 2024).

Efferocytosis failure is *not* merely a defect in apoptotic cell clearance; it represents a mechanistic inflection point at which the plaque transitions from controlled vesicular signaling to uncontrolled release of pro-inflammatory vesicular material. Uncleared late-apoptotic cells progress to secondary necrosis, releasing apoptotic bodies enriched in oxPL, HMGB1, and pro-inflammatory miRNAs that directly fuel the vesicular axis. In this sense, efferocytosis impairment is the key mechanistic bridge linking the mechanotransduction-driven inflammatory milieu to the explosive amplification of EV-mediated signaling—a transition that occupies the centre of the MVDA model developed in Sections 5 and 6.

#### 4.5.1 Molecular mechanisms of phagocytic dysfunction

##### 4.5.1.1 MerTK inactivation

In advanced plaques, pro-inflammatory signals activate ADAM17, which cleaves the MerTK ectodomain to generate soluble sMer; this soluble form acts as a decoy receptor, competitively binding the bridging molecule Gas6 and blocking PS recognition. In Ldlr^−/−^ bone marrow transplant models, introduction of cleavage-resistant MerTK (MerTK^CR) improved efferocytosis efficiency and reduced necrotic core area (Hu et al., 2024). Angiotensin II further promotes MerTK cleavage through the AT1R/ROS/p38 MAPK/ADAM17 pathway, deepening efferocytosis deficiency (Zhang Y. et al., 2019).

##### 4.5.1.2 Upregulation of “don’t-eat-me” signals

CD47 binds SIRPα on phagocyte surfaces and suppresses phagocytic cup formation. Pathological upregulation of CD47 during plaque progression impairs macrophage recognition of apoptotic cells (Yurdagul et al., 2017). Dual blockade of the CD47/SIRPα axis together with ANGPTL3 enhanced efferocytosis and attenuated plaque progression in animal models (Hu et al., 2024) [P].

##### 4.5.1.3 Intrinsic macrophage dysfunction

Chronic lipid overloading induces “phagocytic exhaustion” in lesional macrophages. Lysosomal dysfunction and mitochondrial impairment limit phagosome sealing and phagosome–lysosome fusion, further weakening the capacity for sustained sequential efferocytosis (Yurdagul et al., 2017; Li et al., 2024).

#### 4.5.2 Pathological consequences of secondary necrosis

Uncleared apoptotic cells lose membrane integrity and release intracellular contents, triggering secondary necrosis. The DAMPs that are released—including HMGB1, S100A8/A9, and mitochondrial DNA—activate TLR4, the NLRP3 inflammasome, and the cGAS–STING pathway, generating IL-1β and IL-18 (Tall and Bornfeldt, 2023). Tissue factor and PS enriched in the necrotic core are potent pro-coagulants and can precipitate acute thrombosis once the fibrous cap ruptures. MMPs activated by necrotic material degrade fibrous cap collagen and thin the cap, while CXCL1 and IL-8 recruit additional neutrophils into the plaque (Yurdagul et al., 2017; Li et al., 2024). Each of these consequences then reinforces the upstream defect: an enlarging necrotic core delivers more uncleared apoptotic material, sustains the pro-inflammatory milieu, and progressively drives the lesion toward the vulnerable phenotype.

#### 4.5.3 Crosstalk between efferocytosis deficiency and EV communication

Late-stage apoptotic cells that escape clearance release apoptotic bodies enriched in oxPL, HMGB1, and pro-inflammatory miRNAs. Incomplete internalisation of these apoptotic bodies by efferocytosis-impaired macrophages can trigger pyroptosis or further inflammatory activation, with additional release of NLRP3-activator-laden EVs. Efferocytosis deficiency therefore not only fails to resolve apoptotic load but actively converts that load into a pro-inflammatory vesicular output, sustaining the local inflammatory amplification described in Section 4.5.2 (Wang Z. et al., 2022; Tall and Bornfeldt, 2023) [I].

Therapeutic strategies targeting efferocytosis deficiency are under active investigation, including disruption of CD47/SIRPα signaling to restore “eat-me” recognition (Hu et al., 2024), mitochondria-targeted therapies aimed at improving phagocyte degradative capacity (Li et al., 2024), and nanoparticle-mediated delivery of pro-efferocytic factors such as GAS6 and MFG-E8 to plaque regions. In preclinical models, these approaches have shown capacity to slow necrotic core expansion and reduce plaque destabilisation (Li et al., 2024; Hu et al., 2024); their translational profile is appraised in Section 7.

**FIGURE 3:**
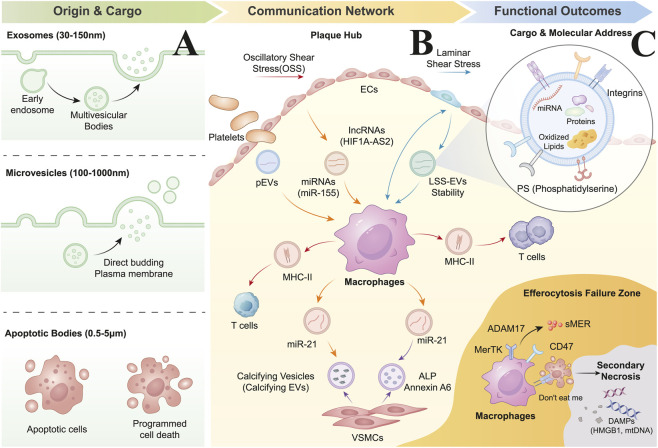
Extracellular Vesicle (EV) Mediated Intercellular Communication and Efferocytosis Impairment in Atherosclerosis. The schematic illustrates the complex vesicular transport network within the plaque microenvironment. **(A)** Biogenesis and Diversity of EVs: Three major types of EVs—exosomes (endosomal origin), microvesicles (plasma membrane budding), and apoptotic bodies (cell fragmentation)—are released by activated endothelial cells (ECs), macrophages, vascular smooth muscle cells (VSMCs), and platelets. **(B)** The Intraplaque Communication Network: EVs serve as functional carriers of miRNAs (e.g., miR-155, miR-21), proteins (e.g., MHC-II, HSPs), and oxidized lipids. Key signaling axes include: (1) EC-to-Macrophage: OSS-induced EVs drive M1 polarization; (2) Macrophage-to-T cell: MHC-II+ EVs present antigens to activate CD4^+^ T cells; (3) Macrophage-to-VSMC: miR-21-rich EVs promote VSMC migration; and (4) VSMC-to-Macrophage: Calcifying EVs (ALP+/Annexin A6+) trigger NLRP3 inflammasome activation. **(C)** Spatial Selectivity and Efferocytosis Failure: The diffusion of EVs is governed by matrix stiffness and specific receptor-ligand pairings (e.g., PS-MerTK). In advanced plaques, efferocytosis is compromised due to MerTK cleavage by ADAM17 and the upregulation of the “do not eat me” signal (CD47). This failure leads to secondary necrosis, the release of DAMPs (e.g., HMGB1), and the expansion of the lipid-rich necrotic core (LRNC), ultimately driving plaque destabilization.

Figure 3 summarises the EV-mediated intercellular communication network and efferocytosis impairment within the atherosclerotic plaque microenvironment. Panel A shows the biogenesis of three major EV subtypes—exosomes, microvesicles, and apoptotic bodies—released by ECs, macrophages, VSMCs, and platelets. Panel B depicts the intraplaque signaling axes carried by EVs, including OSS-induced EC-EVs driving M1 macrophage polarisation, MHC-II^+^ macrophage-EVs activating CD4^+^ T cells, miR-21-rich EVs promoting VSMC migration, and calcifying EVs (ALP^+^/Annexin A6^+^) triggering NLRP3 inflammasome activation. Panel C shows how efferocytosis is progressively impaired in advanced plaques through ADAM17-mediated MerTK cleavage and CD47 upregulation, with secondary necrosis, DAMP release (e.g., HMGB1), and lipid-rich necrotic core expansion as downstream consequences—and identifies several intervention points relevant to the therapies discussed in Section 7.

## 5 Crosstalk between mechanotransduction and vesicular trafficking

### 5.1 Regulation of extracellular vesicle release by mechanical signals

Vascular endothelial cells are continuously exposed to fluid shear stress (FSS), the central mechanobiological stimulus governing vascular homeostasis and the initiation and progression of atherosclerosis (Cheng et al., 2023). Oscillatory shear stress (OSS), which predominates at arterial bifurcations, curvatures, and regions just upstream of stenosis, supports a pro-atherogenic endothelial phenotype, whereas laminar shear stress (LSS) is vasoprotective (He et al., 2022). The two flow patterns act on more than gene expression and phenotype: they also tune EV biogenesis and release through several intracellular signaling cascades (Thompson and Papoutsakis, 2023).

In endothelial cells, OSS activates the small GTPase RhoA, which through ROCK phosphorylates LIM kinase (LIMK) and reorganises the actin cytoskeleton into stress fibres (Power et al., 2024); the resulting cofilin-1 inactivation increases F-actin tension and supports the trafficking of multivesicular bodies (MVBs) toward the plasma membrane, raising exosome output (Davis et al., 2023). Comparative miRNA profiling of endothelial sEVs under LSS versus OSS shows that OSS raises sEV secretion overall and shifts cargo toward pro-inflammatory and pro-angiogenic miRNAs, while LSS-sEVs carry anti-inflammatory species (Chung et al., 2022) [P]. Low shear stress also enhances EV uptake by endothelial cells via MCAM- and PECAM-1-mediated endocytosis, supporting further propagation of pro-inflammatory signals at lesion-prone sites (Coly et al., 2024).

Whether MVBs fuse with lysosomes (for degradation) or with the plasma membrane (releasing exosomes) is a key determinant of total exosome output (van et al., 2018). In macrophages cultured on high-stiffness substrates that mimic the fibrotic plaque environment, nuclear translocation of YAP increases, NF-κB signaling is engaged, lysosomal fusion with MVBs declines, and a greater proportion of MVBs is directed to plasma membrane fusion—raising exosome release substantially (Meli et al., 2020). mTOR participates in this fate switch in two ways: mTORC1 activation upregulates lysosomal biogenesis genes (LAMP1, V-ATPase d2 subunit) and so supports lysosomal degradation of MVBs, whereas mTORC1 inhibition accelerates exosome release in certain cell types (Han et al., 2022). Mechanical signals therefore appear to act bidirectionally on MVB fate through the Akt/mTOR axis, with cell-type- and microenvironment-specific outcomes.

Rab GTPases—including Rab7, Rab11, and Rab27a/b—control MVB transport and docking at the plasma membrane (Han et al., 2022). Mechanical inputs can change the subcellular localisation of these Rab proteins and the recruitment of their effectors by acting on the cytoskeleton, regulating directional exosome secretion. The ESCRT-dependent and ceramide-dependent pathways both contribute to intraluminal vesicle cargo sorting, and mechanical stress can modulate the activity of these pathways to alter exosome cargo composition and release kinetics (Welsh et al., 2024).

### 5.2 Vesicle-mediated transcellular transmission of mechanical signals

EVs are mediators of intercellular communication, but they also “encode” the mechanical state of the parent cell within their cargo, supporting long-distance propagation of mechanically activated signals (Zeng et al., 2024).

Integrins are the principal transmembrane receptors through which cells sense extracellular matrix (ECM) mechanical cues, and their display on the EV surface is one route for transcellular transmission of mechanical states. Mechanically activated endothelial and immune cells can enrich integrins—particularly αv-family and β1 integrins—on the surface of secreted EVs; on uptake, EV-displayed integrins activate the integrin-linked kinase (ILK)–FAK signaling axis and so reconstitute mechanically activated signaling states in distal cells (Multhaupt et al., 2016). Piezo1, a mechanosensitive ion channel expressed in endothelial cells, macrophages, and T cells, senses membrane tension and triggers Ca^2+^ influx (Zhu et al., 2025). Piezo1 activation couples to FAK recruitment and then to calpain-mediated regulation of LFA-1 clustering at the leading edge of migrating T cells, marking Piezo1 as a key node for immune cell mechanosensing (Liu et al., 2024). Cells under mechanical activation can package Piezo1-containing protein fragments into EVs; on uptake by distal cells, these mechanosensor-laden EVs can engage mechanoreceptor signaling cascades in cells that are not themselves subject to mechanical force, supporting transcellular relay of mechanical activation (Mittelheisser et al., 2024).

The miRNA loading profile of endothelial sEVs differs substantially between OSS and LSS: OSS-sEVs are enriched in pro-inflammatory and pro-angiogenic miRNAs targeting inflammatory response, cell migration, and angiogenesis genes, whereas LSS-sEVs are enriched in anti-inflammatory species. These mechanically distinguished sEVs can modify gene expression in distal endothelial cells and induce mechanically responsive gene programmes in cells not directly exposed to fluid shear stress, supporting the concept of EVs as “mechanical state memory units” within the vasculature (Chung et al., 2022).

Within the plaque microenvironment, EVs released in large quantity by dysfunctionally stressed endothelium—particularly at arterial bifurcations under OSS—can be taken up by infiltrating immune cells, delivering pro-inflammatory signals to distal cell populations (Virmani et al., 2006). Exosomal miR-27b-3p secreted by visceral adipocytes activates endothelial NF-κB by downregulating PPARα; reaching distal arterial segments through the circulation, this exemplifies how EVs can carry inflammatory signals to remote vascular sites (Tang et al., 2023). Within the plaque itself, an oxLDL-rich microenvironment leads macrophages to release EVs whose pro-inflammatory cargo activates adjacent endothelial cells and immune cells, supporting a cross-cell-type signal relay circuit (Zhang Y. G. et al., 2019).

The efficiency and specificity of EV-mediated mechanical signal transmission depend on EV surface ligand–receptor recognition, the endocytic route used by the recipient cell (macropinocytosis, clathrin-mediated endocytosis, etc.), and the efficiency of EV cargo release inside the recipient cell (Buzas, 2023). Under inflammatory conditions, plaque-infiltrating cells upregulate integrins, lectins, and adhesion molecules, which can support targeted EV uptake and thereby connect mechanical and inflammatory inputs (Qin et al., 2022).

#### 5.2.1 Evidence stratification

We explicitly frame the concept of EV-mediated transmission of mechanical signals as a forward-looking hypothesis advanced by this review, rather than an established phenomenon (Section 5.2). The supporting evidence stratifies into three tiers: Currently established [E]/[P] — shear-stress-dependent differential EV cargo loading (Chung et al., 2022); integrin and Piezo1 packaging into EV membranes (Multhaupt et al., 2016; Mittelheisser et al., 2024). Plausible inference [I] — EV-mediated reactivation of mechanosensitive transcriptional programmes in distal, mechanically-quiescent recipient cells. Speculative extrapolation [S] — a true “mechanical state memory” that is transferable across vascular compartments. We propose this latter claim as a testable hypothesis awaiting *in vivo* genetic validation (e.g., endothelial-conditional Rab27a/b or Piezo1 knockouts combined with EV tracking), not as an established phenomenon.

### 5.3 Cooperative construction of a spatial gradient in the plaque immune microenvironment

BOX 2 **Formal definition of the Mechano-Vesicular Dual-Axis (MVDA) model.**

The MVDA model is defined as a coupled, bidirectional regulatory system comprising:

(C1) Mechanotransduction axis—conversion of hemodynamic (OSS/LSS) and matrix-stiffness inputs into intracellular signaling via Piezo1, integrins, and YAP/TAZ.
(C2) Vesicular trafficking axis—biogenesis, spatial diffusion, and targeted uptake of EVs carrying mechanically-encoded molecular cargo (mechanosensor fragments, mechanosensitive miRNAs, oxidised lipids).
(C3) Forward coupling (C1 → C2) — mechanical inputs regulate EV biogenesis (RhoA/ROCK-driven MVB transport), cargo composition (mechanosensitive miRNA loading), and release kinetics (mTOR-controlled MVB fate).
(C4) Reverse coupling (C2 → C1) — EV cargo reconstitutes mechanosensitive signaling states in recipient cells, modulating their subsequent mechanical responsiveness (“spatial permeation”).
(C5) Emergent property—establishment of a spatial gradient of immune activation that preferentially concentrates inflammatory output at the plaque shoulder.

The immune microenvironment within atherosclerotic plaques is not homogeneously distributed: cellular composition, molecular signaling states, and ECM biomechanics differ substantially between the plaque shoulder, the periphery of the lipid-rich necrotic core, and the fibrous cap (Wang et al., 2023). The MVDA model defined in Box 2 above is intended to capture how the mechanotransduction and vesicular trafficking axes together construct and maintain this spatial gradient, and how their joint dysregulation drives the transition from stable to vulnerable plaque.

On the mechanotransduction axis (C1), local hemodynamics within the plaque set the type and magnitude of mechanical signals perceived in each region. At plaque shoulder regions, where FSS is low and oscillatory components dominate, endothelial cells engage pro-inflammatory mechanosensing programmes via YAP/TAZ and NF-κB, with endothelial dysfunction as a consequence (Wang et al., 2016). As the plaque progresses, ECM remodeling (increased collagen cross-linking, higher elastic modulus) creates spatial stiffness gradients that, through the integrin–FAK–RhoA axis, are read out as pro-inflammatory and pro-fibrotic transcriptional programmes (Power et al., 2024). Macrophages in stiffer plaque core regions show enhanced inflammatory activation via YAP nuclear translocation, whereas macrophages in softer regions lean toward an M2 phenotype (Mei et al., 2024). This stiffness-driven spatial pattern of macrophage polarisation is one of the main determinants of the local immune gradient.

On the vesicular trafficking axis (C2), EVs spatially integrate and propagate immune signals from cells in different mechanical activation states. Mechanically activated cells within the plaque—OSS-exposed endothelial cells and macrophages on stiff matrix—secrete EVs carrying pro-inflammatory cargo in large quantity. Through local diffusion within the plaque and release into the circulation, these EVs reach cells that are not themselves under mechanical load (Lim and Harraz, 2024). On uptake by VSMCs and resident macrophages in mechanically quiescent regions, pro-inflammatory EVs target immune-regulatory genes through their miRNA cargo (including miR-21 and miR-92a) and partially reconstitute the transcriptional state of the donor cells, carrying pro-inflammatory signals into deeper plaque layers (Jiang et al., 2023).

The two axes are not independent. As defined by C3 and C4 in Box 2, the mechanotransduction axis sets EV biogenesis (output volume and cargo composition), and the vesicular trafficking axis in turn modifies mechanoreceptor expression and cytoskeletal states in recipient cells, altering their sensitivity to subsequent mechanical stimulation (Di et al., 2023). In early plaques, moderate communication between the two axes supports local immune surveillance; in advanced unstable plaques, intensifying OSS and progressive matrix stiffening drive both axes out of balance, with massive release of pro-inflammatory EVs, imbalanced immune cell polarisation, and accumulation of inflammatory signals at the shoulder—ultimately compromising fibrous cap integrity (Yin et al., 2026).

A more speculative aspect of the model concerns whether EVs can effect a “redistribution” of mechanosensing capacity across plaque regions. EVs carrying mechanosensor proteins such as integrins and Piezo1, originating from highly mechanically activated regions, might on uptake confer enhanced mechanical sensitivity on recipient cells in low-stimulus regions—generating a “spatial permeation” effect for mechanical signals (Mukhopadhyay et al., 2024) [S]. This corresponds to C4 in Box 2 and is the locus of the [S]-tier claim defined in Section 5.2.

### 5.4 Key nodal molecules and integrated signal transduction

The mechanotransduction and vesicular trafficking axes share several signaling nodes; the cross-regulation among these nodes is the molecular backbone of the MVDA framework and is the most direct source of candidate therapeutic targets.

#### 5.4.1 KLF2/KLF4 — the flow-sensitive transcriptional gatekeepers

KLF2 and KLF4 are LSS-induced transcription factors that act upstream of, and antagonistically to, the YAP/TAZ and NF-κB axes. Within the MVDA framework, KLF2/4 occupy a distinct regulatory tier: they suppress pro-inflammatory EV biogenesis by transcriptionally repressing NF-κB-driven exosome regulators and by remodeling endothelial metabolism through the KLF4–GCKR–AMPK axis (Section 3.2.4) (Han et al., 2021). Loss of KLF2/4 — for example, through γ-protocadherin-mediated suppression (Joshi et al., 2024) — therefore de-represses the entire mechano-vesicular cascade. With KLF2/4 inserted as the upstream flow-sensitive gate, the nodal hierarchy of the MVDA framework becomes: KLF2/4 (upstream gate) → YAP/TAZ + NF-κB (parallel effectors) → PI3K/Akt/mTOR (vesicular output controllers).

YAP/TAZ (Yes-associated protein and the transcriptional coactivator with PDZ-binding motif) are the core transcriptional coactivators of the Hippo pathway and the main mechanical effector molecules in vascular mechanotransduction. In endothelial cells, OSS drives YAP/TAZ nuclear translocation and upregulates target genes including CTGF, CYR61, and ANKRD1, supporting a pro-inflammatory phenotype (Jiang et al., 2023); LSS, by contrast, activates Hippo signaling and so phosphorylates LATS1/2 and YAP/TAZ, sending YAP/TAZ for cytoplasmic degradation and maintaining endothelial homeostasis (Ritsvall and Albinsson, 2024). In macrophages, matrix stiffness regulates YAP nuclear translocation through the integrin–RhoA–ROCK axis, and YAP activation together with NF-κB drives expression of IL-1β and TNF-α (Meli et al., 2020). The activity state of YAP/TAZ also modulates MVB transport efficiency and exosome release by tuning actin polymerisation and Rab GTPase recruitment, so that mechanical input is in part translated into changes in EV output (Ritsvall and Albinsson, 2024) [P].

NF-κB is the central inflammatory pathway in plaque. OSS and high matrix stiffness activate NF-κB via the integrin–IKK axis and Piezo1-mediated Ca^2+^ influx, driving expression of ICAM-1, VCAM-1, and pro-inflammatory chemokines in endothelial cells (Lim and Harraz, 2024). NF-κB activation upregulates exosome biogenesis-related genes and, through MCP-1 and IL-6 secretion, shapes the recruitment and activation of monocyte–macrophage populations—and so indirectly sets the cellular sources and cargo composition of plaque EVs (Yin et al., 2026). Endothelial-cell-derived exosomes that carry NF-κB-activating proteins or stimulatory miRNAs can in turn engage NF-κB in recipient macrophages, providing a transcellular route by which the endothelial inflammatory signal is amplified within the macrophage compartment (Wang et al., 2023) [P].

The PI3K/Akt pathway connects mechanical inputs to cellular metabolism and EV biogenesis. Mechanical stimuli activate PI3K through the integrin–FAK axis, leading to Akt phosphorylation. Activated Akt then regulates MVB fate by phosphorylating TSC1/2 to activate mTORC1, modulates MVB cytoplasmic transport through RhoA/ROCK-driven cytoskeletal reorganisation, and supports cell viability by suppressing pro-apoptotic proteins (Bad, Caspase-9), maintaining a sustained exosome secretory programme (Iranpanah et al., 2023). PI3K/Akt activity also affects integrin activation state and surface expression, and through this it sets the integrin display density on EVs and the targeting specificity of EVs for recipient cells (Multhaupt et al., 2016) [P].

The mTOR signaling network, downstream of PI3K/Akt, integrates extracellular mechanical signals with intracellular metabolic state to set exosome biogenesis. mTORC1 controls MVB fate between lysosomal degradation and plasma membrane fusion by regulating lysosomal biogenesis factors (TFEB nuclear translocation and related transcription factors) (Han et al., 2022); mTORC2 affects cytoskeletal dynamics and cell survival through Akt phosphorylation at Ser473. The local metabolic microenvironment of the plaque—lipid accumulation and hypoxia—adds a metabolic input on top of the mechanical one, so that mechanical, metabolic, and vesicular regulation converge at mTOR. Caveolin-1 (CAV1) supports exosome secretion by keeping the PI3K/Akt/mTOR pathway active and suppressing autophagic degradation of MVBs; it also modulates M2 polarisation of macrophages (Zhai et al., 2026) [P].

These nodes form the connective tissue of the MVDA framework. KLF2/4 set the upstream flow-sensitive gate (Section 3.2.2); YAP/TAZ and NF-κB are the parallel transcriptional effectors that integrate mechanical and inflammatory inputs; PI3K/Akt and mTOR control MVB fate and the EV output that propagates the signal to distal recipient cells. This nodal hierarchy is what Box 2 designates as forward and reverse coupling (C3, C4); the resulting spatial gradient of immune activation at the plaque shoulder is the emergent property C5 (Han et al., 2025).

**FIGURE 4:**
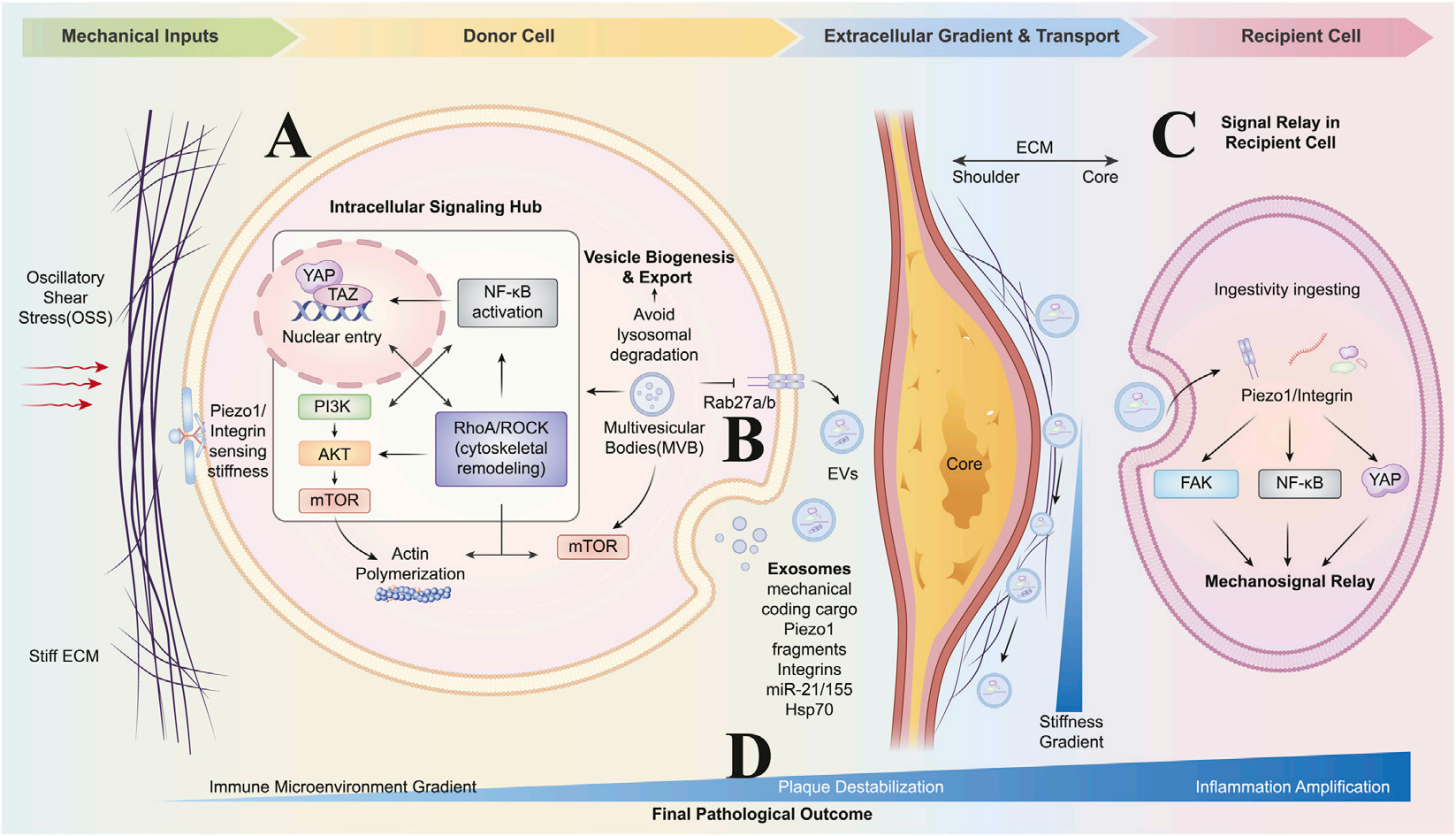
The Mechano-Vesicular Dual-Axis Model in the Orchestration of Plaque Immune Microenvironment. This integrative schematic illustrates the synergistic crosstalk between mechanotransduction and extracellular vesicle (EV) trafficking in atherosclerosis. **(A)** Mechanically Driven EV Biogenesis: Pro-atherogenic mechanical cues, such as oscillatory shear stress (OSS) and high matrix stiffness, activate primary mechanosensors (Piezo1, Integrins). These signals converge on a central regulatory hub comprising the RhoA/ROCK, YAP/TAZ, NF-κB, and PI3K/Akt/mTOR pathways. This signaling network promotes the transport of multivesicular bodies (MVBs) toward the plasma membrane while inhibiting their lysosomal degradation, leading to increased secretion of EVs. **(B)** Encoding of “Mechanical Memory”: EVs secreted from mechanically activated cells are enriched with “mechanically responsive” cargoes, including mechanosensitive ion channel fragments (Piezo1), adhesion receptors (Integrins), and pro-inflammatory miRNAs (e.g., miR-155, miR-21). **(C)** Spatial Signal Relay and Amplification: These EVs diffuse through the spatially heterogeneous plaque matrix, guided by stiffness gradients. Upon uptake by distant recipient cells (e.g., macrophages, VSMCs in quiescent zones), the EV-encapsulated “mechanical codes” trigger a mechanosensitive-like signaling cascade (e.g., FAK and YAP activation) in the absence of direct physical force. **(D)** Spatial Gradient Formation: The coupling of the mechanotransduction axis and the vesicle transport axis establishes a spatial gradient of immune activation, preferentially concentrating inflammatory signals at the plaque shoulder and driving the transition from a stable to a vulnerable plaque phenotype.

Figure 4 summarises the MVDA model that integrates mechanotransduction and EV trafficking as cooperative drivers of plaque immune microenvironment organisation. Panel A shows how pro-atherogenic mechanical cues (OSS, high matrix stiffness) activate Piezo1 and integrins, converging on RhoA/ROCK, YAP/TAZ, NF-κB, and PI3K/Akt/mTOR to support MVB exocytosis and increased EV secretion (corresponding to C1 and forward coupling C3 in Box 22). Panel B shows that these mechanically activated EVs carry encoded “mechanical memory” in the form of Piezo1 fragments, adhesion receptors, and pro-inflammatory miRNAs (miR-155, miR-21). Panel C depicts how recipient cells in mechanically quiescent zones internalise these EVs and recapitulate mechanosensitive-like signaling (FAK, YAP activation) without direct physical stimulation—the reverse coupling C4. Panel D synthesises these axes, showing how their coupling establishes the spatial gradient of immune activation (C5) that concentrates inflammatory signals at the plaque shoulder and supports the transition from stable to vulnerable phenotype.

## 6 Spatial architectural disruption and plaque vulnerability

### 6.1 Comparative spatial immunological profiles of stable versus vulnerable plaques

Table 3 summarises the mechano-vesicular-immune triad across the dimensions that distinguish stable from vulnerable plaques, together with the principal unresolved questions and candidate therapeutic levers for each. The clinical fate of atherosclerotic plaques—long-term stability or acute rupture—is governed largely by the structural integrity of the intra-plaque spatial architecture and the functional balance of its immune microenvironment. Spatial transcriptomics, single-cell RNA sequencing, and multimodal imaging now allow systematic comparison of stable and vulnerable plaques across three dimensions: the mechanical microenvironment, the EV secretory profile, and the spatial distribution of immune cells.

**TABLE 3:** The mechano-vesicular-immune triad: dimensional comparison of stable and vulnerable plaques.

Dimension	Stable plaque	Vulnerable plaque	Unresolved question	Therapeutic lever	Key evidence
Mechanical	Homogeneous LSS; uniform stiffness	OSS at shoulder; stiffness mismatch from microcalcifications	*In vivo* dynamic mapping of plaque stress fields	Hemodynamic optimisation; KLF2/4 induction	Shinge et al. (2022), Jansen et al. (2024), Corti et al. (2022), Kong et al. (2022)
Vesicular	miR-146a-rich anti-inflammatory EVs	miR-155/oxPL pro-inflammatory EVs	Cell-of-origin *in vivo* EV tracking	Engineered M2-EVs; anti-miR-155 therapeutics	He et al. (2018), Patel et al. (2023)
Immune	Ordered Treg/TREM2^+^ topology	Shoulder-infiltrating M1/CD8^+^/Th1	Causality of redistribution (drive vs. consequence)	Spatially-targeted immunomodulation	Wolf and Ley (2019), Fernandez et al. (2019), Kyaw et al. (2013)

EV, extracellular vesicle; LRNC, lipid-rich necrotic core; LSS, laminar shear stress; OSS, oscillatory shear stress; oxPL, oxidised phospholipids; SMC, smooth muscle cell; TCFA, thin-cap fibroatheroma; TREM2, triggering receptor expressed on myeloid cells 2; Treg, regulatory T cell. Numbers in the final column refer to references in the reference list.

#### 6.1.1 Mechanical microenvironment

Stable plaques have a thick, structurally intact fibrous cap with well-organised type I collagen, a relatively homogeneous elastic modulus, and a stable local shear stress distribution. Under sustained LSS, endothelial cells upregulate KLF2 and KLF4, which activate endothelial nitric oxide synthase (eNOS) and suppress NF-κB signaling, downregulating adhesion molecules including ICAM-1 and VCAM-1 and reducing transendothelial monocyte migration (Joshi et al., 2024; Chiu et al., 2011). The protective role of the KLF2/KLF4 axis has been confirmed in experimental and human models, and targeted modulation of this axis is a candidate anti-atherosclerotic strategy (Joshi et al., 2024).

Vulnerable plaques—TCFAs—typically have a fibrous cap below 65 μm, sparse SMCs, marked ECM degradation, and pronounced heterogeneity of local elastic modulus: stiffness in the peri-necrotic core region can exceed that of normal vascular wall by orders of magnitude, while the cap–shoulder junction is abnormally compliant owing to severe collagen depletion (Jansen et al., 2024; Corti et al., 2022). This stiffness discontinuity generates localised stress concentrations under cyclical blood pressure loading, and recurrent OSS at the plaque shoulder sustains pro-inflammatory endothelial signaling. These features establish the biomechanical substrate of rupture susceptibility, but should not be read deterministically: spatial heterogeneity is a necessary, not sufficient, condition for rupture. Many spatially disorganised plaques remain clinically silent over decades, indicating that additional triggering factors (acute hemodynamic stress, systemic inflammatory flares) are needed to precipitate an event (Shinge et al., 2022; Kong et al., 2022).

#### 6.1.2 Extracellular vesicle secretory profile

Stable and vulnerable plaques have distinct EV secretory signatures. Within the stable plaque microenvironment, anti-inflammatory EVs predominate: M2-polarised macrophage exosomes are enriched in anti-inflammatory miRNAs such as miR-146a, while EVs from homeostatic endothelial cells carry elevated eNOS protein and support endothelial homeostasis through paracrine signaling (Patel et al., 2023). In vulnerable plaques, the EV repertoire shifts: oxidative stress and hypoxia drive endothelial cells and M1 macrophages to release exosomes enriched in pro-inflammatory miRNAs such as miR-155, and pro-inflammatory microvesicles loaded with oxidised phospholipids (oxPL) (Patel et al., 2023) [P].

Accumulation of apoptotic bodies around the necrotic core of vulnerable plaques is a direct consequence of impaired efferocytosis; the oxPL cargo of these bodies activates the NLRP3 inflammasome in neighbouring macrophages and reinforces the local inflammatory response (Doran et al., 2020; Swanson et al., 2019). Proteomic and microRNA profiling of circulating EVs has been proposed as a non-invasive biomarker strategy for distinguishing stable from vulnerable plaque states. Circulating monocyte-derived EV levels are significantly higher in acute coronary syndrome (ACS) than in stable coronary artery disease, and this difference tracks the degree of plaque inflammatory activation (Loyer et al., 2018). EVs also carry MMP-9 and tissue factor (TF)-associated proteins, raising the possibility that EVs are not only carriers of inflammatory signals but contributors to secondary thrombosis after plaque rupture (Patel et al., 2023).

#### 6.1.3 Spatial distribution of immune cells

Immune cells in stable plaques are arranged with relatively ordered compartmentalisation: TREM2^+^ and CD163^+^ M2-like macrophages localise to the periphery of the plaque core and around neovessels, performing lipid scavenging; regulatory T cells (Tregs) maintain a significant presence beneath the fibrous cap and at the shoulder margins, supporting local immunosuppressive homeostasis through IL-10 and TGF-β (Wolf and Ley, 2019; Depuydt et al., 2020; Patterson et al., 2023). Single-cell sequencing has shown high spatial co-localisation between TREM2^+^ macrophages and fibrous cap SMCs in stable plaques, indicating that the two populations cooperate to preserve cap structural integrity (Patterson et al., 2023).

Vulnerable plaques show a strikingly different immune cell landscape. Pro-inflammatory macrophages—high TNF, IL-1β, MMP-9 — are markedly increased in proportion and absolute number, with aberrant infiltration into the fibrous cap and shoulder regions (Fernandez et al., 2019). Elevated CD8^+^ cytotoxic T cell density at the shoulder correlates positively with plaque rupture risk; these cells induce SMC apoptosis through perforin and granzyme B, eroding fibrous cap structural integrity (Kyaw et al., 2013). Treg depletion and a Th1/Th17 shift are characteristic of immune dysregulation in vulnerable plaques (Wolf and Ley, 2019; Tabas et al., 2020). Single-cell analyses also show pronounced reciprocal activation between pro-inflammatory macrophages and T cells in vulnerable plaques—a cellular dialogue that is constrained by Treg-mediated suppression in stable plaques (Depuydt et al., 2020; Zernecke et al., 2023).

##### 6.1.3.1 Unified three-dimensional synthesis

Stable and vulnerable plaques can be distinguished by a single integrated criterion: the coherence of the mechano-vesicular-immune triad. Stable plaques show coherent triad alignment—homogeneous LSS, an anti-inflammatory EV repertoire, and compartmentalised immune topology. Vulnerable plaques show triad disorganisation—heterogeneous OSS/stiffness, pro-inflammatory EV output, and aberrant immune cell invasion of structurally critical zones. It is the triad’s coherence, rather than any single dimension in isolation, that governs plaque fate (see Table 3).

### 6.2 Triggering mechanisms of mechanical destabilisation

The mechanical integrity of the fibrous cap is the final barrier against plaque rupture, and its thickness and tensile strength depend on SMC viability, the balance between collagen synthesis and degradation, and overall mechanical loading. When this equilibrium is disturbed, several layered pathological processes are activated in sequence.

#### 6.2.1 SMC apoptosis and fibrous cap structural weakening

Fibrous cap SMCs continuously synthesise type I and type III collagen and constitute the cellular foundation of cap tensile strength. In vulnerable plaques, SMC number and function are compromised by combined mechanical and inflammatory signals. OSS activates the mechanosensitive ion channel Piezo1 on SMCs, leading to intracellular Ca^2+^ overload and apoptotic signaling (Shinge et al., 2022); pathologically elevated matrix stiffness drives SMC transdifferentiation toward an osteogenic phenotype through the YAP/TAZ–Hippo axis, with loss of collagen-synthesising function (Wang et al., 2020). Lineage tracing and single-cell sequencing show that a subset of fibrous cap SMCs undergoes phenotypic switching during plaque progression, transdifferentiating into macrophage-like cells with phagocytic activity but no collagen-synthesising capacity, further compromising cap strength (Alencar et al., 2020).

On the inflammatory side, infiltrating CD8^+^ T cells lyse fibrous cap SMCs through the perforin–granzyme pathway, while M1 macrophages drive SMC apoptosis through pro-inflammatory cytokine secretion (Kyaw et al., 2013; Tabas et al., 2020). As the principal effector cytokine of Th1 cells, IFN-γ suppresses COL1A1 and COL3A1 transcription in SMCs, reducing collagen synthesis (Wolf and Ley, 2019). Apoptotic bodies released by apoptotic SMCs that are not promptly cleared carry mitochondrial DNA capable of activating innate immune pathways in neighbouring macrophages, amplifying local inflammatory signals (Doran et al., 2020; Kumar et al., 2023). EVs also contribute to SMC functional impairment: pro-inflammatory miRNA-enriched exosomes released by M1 macrophages suppress SMC proliferation and accelerate apoptosis on uptake (Patel et al., 2023).

The heterogeneity of the SMC lineage is closely linked to plaque stability. KLF4-dependent phenotypic switching pushes a subset of SMCs into “fibromyocyte-like” or “macrophage-like” states, with consequences for both plaque inflammation and structural stability (Alencar et al., 2020; Tang H. Y. et al., 2022); osteogenic SMC transdifferentiation contributes directly to plaque calcification through matrix vesicle secretion (Jinnouchi et al., 2020).

#### 6.2.2 Collagen degradation and the MMP activation loop

In the normal vascular wall, MMP activity is tightly regulated by tissue inhibitors of metalloproteinases (TIMPs), with the two systems in dynamic equilibrium. In the chronic inflammatory microenvironment of vulnerable plaques, activated macrophages secrete abundant MMP-1, MMP-8, MMP-9, and MMP-13, while TIMP-1 and TIMP-2 expression is markedly downregulated (Jansen et al., 2024; Samah et al., 2023). These MMPs degrade type I and type III collagen within the fibrous cap, lowering tensile strength and exposing deep tissue factor as a pro-thrombotic substrate (Jansen et al., 2024; Alonso-Herranz et al., 2023). Clinical evidence indicates that MMP-2 activity is significantly elevated in patients with unstable compared with stable coronary artery disease, supporting MMP system imbalance as a molecular determinant of plaque instability (Samah et al., 2023).

OSS upregulates MMP-9 secretion in endothelial cells through NF-κB activation, while elevated matrix stiffness promotes MMP-12 transcription in macrophages through integrin signaling, degrading elastin within the fibrous cap and surrounding matrix (Shinge et al., 2022; Kong et al., 2022). As MMP-mediated ECM degradation proceeds, the local mechanical properties of the fibrous cap deteriorate, and the resulting aberrant mechanical signaling reinforces MMP upregulation—so that proteolysis and mechanical destabilisation drive one another rather than acting in sequence [P].

##### 6.2.2.1 EVs as multi-channel amplification nodes

EVs are not auxiliary participants but active signal amplifiers within this destabilisation loop. Vulnerable-plaque-derived microvesicles at the same time, deliver MMP-associated proteins, pro-inflammatory miRNAs, and surface-displayed mechanosensors to recipient cells, coupling proteolytic, transcriptional, and mechanosensitive outputs in a single transmission event. Each EV uptake event therefore functions as a multi-channel amplification node, not a downstream consequence—a positioning consistent with the MVDA model’s reverse coupling (Box 2, C4). Microvesicles released by vulnerable plaques carry MMP-associated proteins and pro-inflammatory miRNAs that, on uptake by target cells, promote MMP-9 transcription through the PI3K/Akt pathway (Patel et al., 2023), directly translating the C4 reverse coupling into a measurable proteolytic readout.

#### 6.2.3 Local stress concentration and the bidirectional effects of microcalcification

As the fibrous cap thins and ECM degradation intensifies, plaque mechanical destabilisation enters an accelerated phase. Computational mechanics studies indicate that when fibrous cap thickness falls below a critical threshold, maximum circumferential stress within the cap rises sharply, approaching or exceeding the tensile failure limit of collagen fibre bundles (Jansen et al., 2024). The stress concentration effect is most pronounced at the plaque shoulder, a region subjected to hemodynamic shear stress and radial tensile forces from pulsatile blood pressure, and the site of densest M1 macrophage and T cell infiltration (Jansen et al., 2024; Alonso-Herranz et al., 2023).

Plaque calcification has bidirectional effects on fibrous cap integrity. Diffuse sheet-like calcification increases overall plaque stiffness and may confer some structural stability; punctate microcalcifications (diameter 5–65 μm) at the periphery of the necrotic core, however, generate local stress amplification owing to stiffness discontinuity with the surrounding soft tissue, raising rupture risk (Corti et al., 2022; Kawtharany et al., 2022; Montanaro et al., 2021). The spatial distribution of microcalcifications—rather than total burden—is the critical determinant of their effect on stability: clustered microcalcifications within the fibrous cap produce greater stress concentration than single larger calcific deposits (Kawtharany et al., 2022). Inflammation and microcalcification feed one another: activated macrophages support microcalcification formation through secretion of pro-inflammatory mediators and related enzymes, while microcalcifications themselves induce local cell necrosis and further inflammation through mechanical mechanisms (Kawtharany et al., 2022).

Matrix vesicles released by apoptotic SMCs are the principal initiators of hydroxyapatite crystal nucleation within plaques; their phospholipid bilayers are enriched in phosphatidylserine, providing a nucleation template for calcium-phosphate crystallisation (Jinnouchi et al., 2020). Elevated matrix stiffness activates osteogenic transcriptional programmes—including Runx2 — through YAP nuclear translocation, leading SMCs to release pro-calcific matrix vesicles in larger quantity; the resulting calcification raises local stiffness further, so that established osteogenic transformation in SMCs tends to sustain itself once initiated (Wang et al., 2020; Tang H. Y. et al., 2022). The mechanical destabilisation circuit in 6.2 is therefore best read as a coupled mechano-cellular-molecular system in which EV-mediated transmission (Box 2, C3/C4) serves as the principal relay.

### 6.3 Immune cell spatial redistribution and plaque rupture

Plaque rupture is not a stochastic mechanical event but the culmination of aberrant immune cell accumulation at specific anatomical sites—most in particular the shoulder region—superimposed on progressive local structural destruction. Understanding this process requires integrated analysis of both mechanical triggering and vesicle-mediated mechanisms.

#### 6.3.1 Mechanical triggering of aberrant immune cell accumulation at the shoulder region

The plaque shoulder is a transitional zone between plaque boundary and normal vascular wall, characterised by the high-density convergence of four spatial features: (1) peak oscillatory shear stress; (2) the thinnest fibrous cap and most loosely organised ECM; (3) the highest density of macrophages and T cells; and (4) the highest MMP activity. The spatial co-occurrence of these features makes the shoulder the most structurally vulnerable site within the plaque (Jansen et al., 2024; Libby et al., 2019).

Mechanical regulation of directed immune cell migration toward the shoulder operates at multiple levels. OSS at the shoulder activates endothelial NF-κB, upregulating CXCL1 and CXCL8, and ICAM-1 and VCAM-1, providing spatial recruitment signals for circulating monocytes (Chiu et al., 2011; Kong et al., 2022). The stiffness gradient of shoulder ECM directs macrophages that have infiltrated the plaque to migrate toward stiffer regions via durotaxis; this migratory path traverses the most mechanically compromised zone of the fibrous cap, contributing mechanical damage as well as inflammation (Atcha et al., 2021). Piezo1 channel-mediated mechanosignaling modulates macrophage polarisation state and migration velocity: myeloid-specific Piezo1 deletion reduces macrophage infiltration in atherosclerotic mouse plaques, while excessive Piezo1 activation aggravates plaque inflammation through enhanced oxLDL uptake and pro-inflammatory polarisation (Atcha et al., 2024).

Aberrant T cell accumulation at the shoulder also has a mechanical driving component. CD4^+^ Th1 cells dominate at the TCFA shoulder, and their local concentration tracks IFN-γ secretion, which inhibits SMC collagen synthesis and enhances macrophage pro-inflammatory activity (Wolf and Ley, 2019; Tabas et al., 2020). OSS upregulates endothelial CXCL10 secretion, supporting directed Th1 cell recruitment; elevated matrix stiffness raises local T cell activation by enhancing MHC class II presentation efficiency on antigen-presenting cells (Kong et al., 2022). CD8^+^ T cells induce fibrous cap SMC apoptosis through the perforin–granzyme B pathway, contributing to the progressive weakening of plaque structural integrity (Kyaw et al., 2013).

#### 6.3.2 Vesicle-mediated spatial redistribution of immune cells

EVs play two complementary roles in immune cell spatial redistribution: “remote recruitment” and “*in situ* polarisation.” Highly activated pro-inflammatory cells in the plaque shoulder release EVs in large quantity; these vesicles disseminate via blood flow or permeate through ECM interstitial spaces, transmitting inflammatory activation signals to circulating monocytes and T cells, upregulating chemokine receptor expression on these cells, and supporting their directional homing to high-inflammation zones within the plaque (Patel et al., 2023). This “remote broadcasting” mechanism transcends the distance constraints of local paracrine signaling, allowing the plaque inflammatory microenvironment to recruit effector immune cells from the systemic circulation (Patel et al., 2023).

Immune cells that have reached the shoulder are then subject to continuous functional remodeling by locally produced EVs. Pro-inflammatory exosomes released by M1 macrophages, on uptake by neighbouring TREM2^+^ or M2-like macrophages, reprogramme these cells toward a pro-inflammatory phenotype, gradually eroding the anti-inflammatory stabilising macrophage population at the shoulder (Patel et al., 2023; Patterson et al., 2023). The hypoxic shoulder microenvironment drives macrophage secretion of HIF-1α-associated exosomes; on uptake by Treg cells, these EVs suppress FoxP3 stability, leading to Treg functional impairment and apoptosis, and further disturbing local immunosuppressive homeostasis (Wolf and Ley, 2019).

Damage-associated molecular patterns (DAMPs) released by apoptotic bodies that accumulate as a consequence of impaired efferocytosis activate local innate immune cells and support further pro-inflammatory cytokine secretion; as the apoptotic load grows, innate immune activation in turn supports adaptive immune amplification, but the sequence is not a simple cascade—each step feeds back to the others (Doran et al., 2020; Kumar et al., 2023). The NLRP3 inflammasome is central to the maintenance and amplification of plaque inflammation: cholesterol crystals and oxLDL activate NLRP3, driving macrophage secretion of IL-1β and reinforcing the inflammatory state of the shoulder (Duewell et al., 2010; Swanson et al., 2019).

### 6.4 Quantitative associations between spatial redistribution and plaque rupture risk

Multiple studies using pathological anatomy as the gold standard have validated quantitative associations between immune cell spatial redistribution and plaque rupture. Macrophage density at the shoulder of ruptured plaques is several-fold higher than in stable plaques, and the spatial proximity between the macrophage infiltration front and the thinnest point of the fibrous cap is a critical pathological prerequisite for rupture (Jansen et al., 2024; Libby et al., 2019). Single-cell sequencing has shown that ACS plaques harbour an “inflammation–degradation co-expression gene module” — high spatial co-localisation of IL-1β, MMP-9, CXCL8 and related molecules—that is absent in stable plaques (Emoto et al., 2022).

The spatial activation pattern of the NLRP3 inflammasome is directly associated with rupture risk: NLRP3-activated macrophage density at the shoulder of ruptured plaques is significantly higher than in the central fibrous cap region, and the degree of NLRP3 activation tracks local IL-1β concentration and MMP-9 activity (Duewell et al., 2010; Fidler et al., 2021). In the CANTOS trial, anti-inflammatory therapy targeting IL-1β reduced the risk of major adverse cardiovascular events, supporting the position that inflammatory activation at the plaque shoulder is a central driver of cardiovascular events (Ridker et al., 2017). Single-cell sequencing of ACS culprit plaques has shown that, compared with stable plaques, myeloid-derived cells in ACS culprit plaques exhibit more pronounced pro-inflammatory activation (Emoto et al., 2022) — so the functional state of immune cells, not just their quantity, is an important parameter for predicting plaque vulnerability [P].

Taking the mechanical triggering and vesicle-mediated processes together, the spatial redistribution of immune cells toward the shoulder reflects a coupling between mechanics, EV signaling, and immune cell activation in which each component sustains the others rather than acting in series. The end point of this coupled process is fibrous cap rupture, exposure of the deep lipid core and tissue factor, and acute intraluminal thrombus formation—precipitating events including acute myocardial infarction and unstable angina (Libby et al., 2019; Ridker et al., 2017).

Recent human plaque spatial atlases—including high-resolution carotid and coronary single-cell + spatial transcriptomic integrations—have begun to map cell–cell communication networks within native human plaque microenvironments at unprecedented resolution. We here assess how the MVDA model aligns with, and where it diverges from, these human datasets.

Convergent findings (human-validated [E]/[P]). (i) Spatial co-localisation of inflammatory macrophage subsets with shoulder-region OSS exposure is consistent across high-resolution human carotid plaque atlases (Depuydt et al., 2020; Mosquera et al., 2023). (ii) KLF2/4-defined endothelial subset zonation under flow has been confirmed in human samples (Joshi et al., 2024). (iii) TREM2^+^ macrophage localisation at the LRNC periphery is reproducible across independent human single-cell studies (Jaitin et al., 2019). Together, these findings substantiate the mechanotransduction axis (C1) and the emergent spatial immune topology (C5) of the MVDA model in human tissue.

Murine-only findings requiring human confirmation [P]. (i) Piezo1 myeloid-specific deletion phenotypes (Atcha et al., 2024) have not been reproduced in human conditional models, which remain impossible for ethical reasons; surrogate readouts from human PIEZO1 haploinsufficient carriers are scarce. (ii) MerTK^CR rescue of efferocytosis (Cai et al., 2017) has been validated in murine bone marrow transplant models but not in human plaque tissue. (iii) Engineered M2-EV plaque biodistribution and therapeutic efficacy (Xie et al., 2024) are documented in ApoE^−/−^ models; large-animal and human imaging-based biodistribution data are needed before translation.

Currently unvalidated in either species [I]/[S]. (i) EV-mediated transcellular transmission of mechanical state *in vivo* remains principally inferential—supported by *in vitro* cargo-loading and uptake data but not by donor-to-recipient EV tracking in intact tissue. (ii) MVDA reverse-coupling causality (Box 2, C4) — the claim that EV cargo reconstitutes mechanosensitive signaling in mechanically quiescent recipient cells *in vivo*—has no genetic-level causal validation in either murine or human tissue at the time of this review.

Read together, the MVDA model’s mechanotransduction axis and spatial immune topology are substantially human-validated, whereas its vesicular reverse-coupling claims remain principally murine and *in vitro*. The decisive next step is human *ex vivo* plaque imaging-mass cytometry combined with EV phenotyping—a methodologically demanding but tractable programme that would directly graduate C4 from [I] to [E]. Until that evidence accumulates, the MVDA model is best read as a hybrid framework: a well-validated mechano-immune scaffold (C1, C5) supporting a forward-looking vesicular hypothesis (C2, C3, C4).

### 6.5 Cross-validation against emerging human plaque spatial atlases

Recent human plaque spatial atlases—including high-resolution carotid and coronary single-cell + spatial transcriptomic integrations—have begun to map cell–cell communication networks within native human plaque microenvironments at unprecedented resolution. We here assess how the MVDA model aligns with, and where it diverges from, these human datasets. The full evidence stratification of all key mechanistic claims is summarised in Table 1.

1. Convergent findings (human-validated [E]/[P]) (i) Spatial co-localisation of inflammatory macrophage subsets with shoulder-region OSS exposure is consistent across high-resolution human carotid plaque atlases (Depuydt et al., 2020; Mosquera et al., 2023). (ii) KLF2/4-defined endothelial subset zonation under flow has been confirmed in human samples (Joshi et al., 2024). (iii) TREM2^+^ macrophage localisation at the LRNC periphery is reproducible across independent human single-cell studies (Jaitin et al., 2019). Together, these findings substantiate the mechanotransduction axis (C1) and the emergent spatial immune topology (C5) of the MVDA model in human tissue.
2. Murine-only findings requiring human confirmation [P] (i) Piezo1 myeloid-specific deletion phenotypes (Atcha et al., 2024) have not been reproduced in human conditional models, which remain impossible for ethical reasons; surrogate readouts from human PIEZO1 haploinsufficient carriers are scarce. (ii) MerTK^CR rescue of efferocytosis (Cai et al., 2017) has been validated in murine bone marrow transplant models but not in human plaque tissue. (iii) Engineered M2-EV plaque biodistribution and therapeutic efficacy (Xie et al., 2024) are documented in ApoE^−/−^ models; large-animal and human imaging-based biodistribution data are needed before translation.
3. Currently unvalidated in either species [I]/[S] (i) EV-mediated transcellular transmission of mechanical state *in vivo* remains principally inferential—supported by *in vitro* cargo-loading and uptake data but not by donor-to-recipient EV tracking in intact tissue. (ii) MVDA reverse-coupling causality (Box 2, C4) — the claim that EV cargo reconstitutes mechanosensitive signaling in mechanically quiescent recipient cells *in vivo*—has no genetic-level causal validation in either murine or human tissue at the time of this review.

Read together, the MVDA model’s mechanotransduction axis and spatial immune topology are substantially human-validated, whereas its vesicular reverse-coupling claims remain principally murine and *in vitro*. The decisive next step is human *ex vivo* plaque imaging-mass cytometry combined with EV phenotyping—a methodologically demanding but tractable programme that would directly graduate C4 from [I] to [E]. Until that evidence accumulates, the MVDA model is best read as a hybrid framework: a well-validated mechano-immune scaffold (C1, C5) supporting a forward-looking vesicular hypothesis (C2, C3, C4).

**FIGURE 5:**
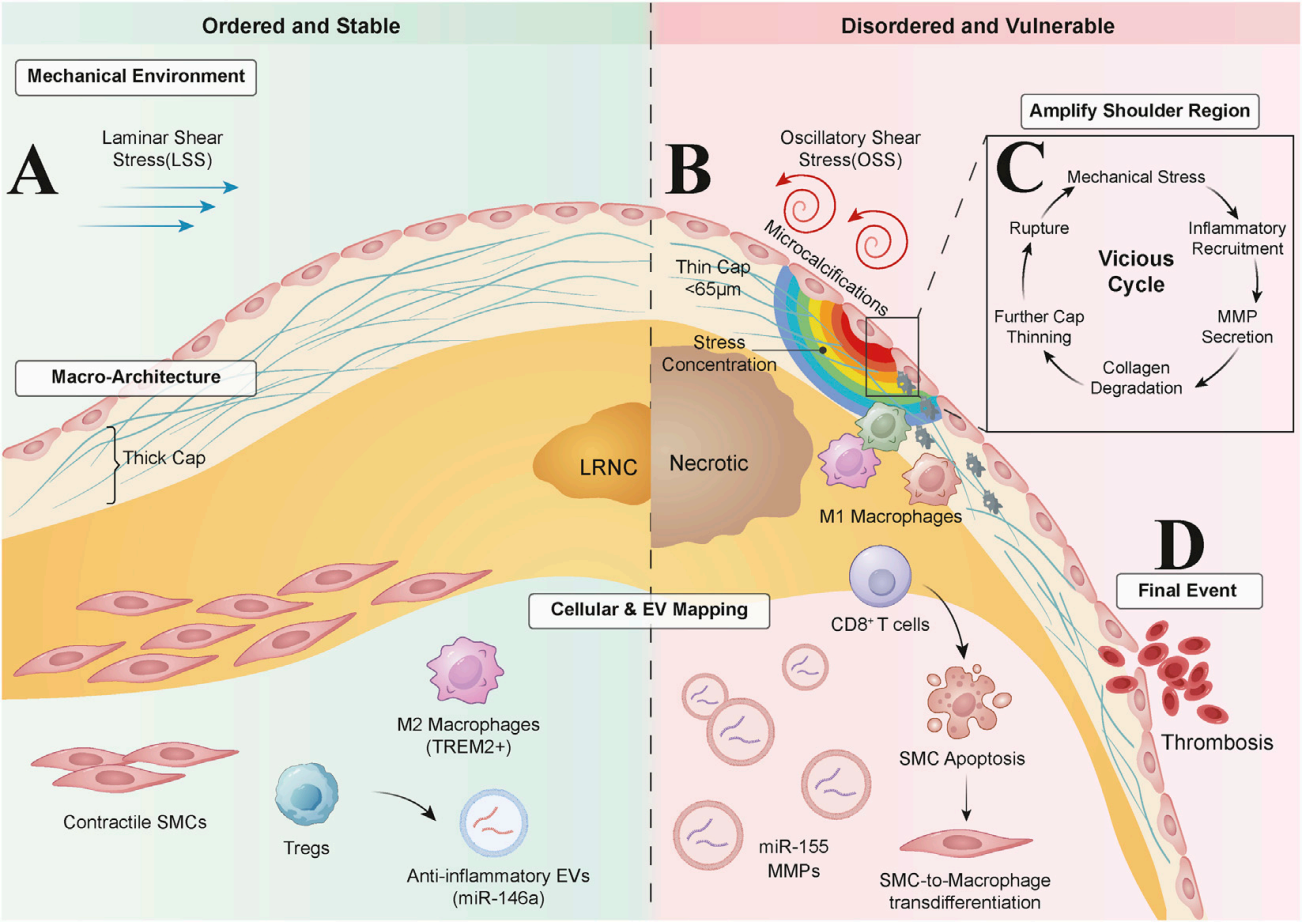
Spatial Disorganization and the Transition from Stable to Vulnerable Plaque. The schematic provides a multi-dimensional comparison between stable and vulnerable atherosclerotic plaques, highlighting the role of spatial architecture in plaque rupture. **(A)** Stable Plaque: Characterized by a thick fibrous cap, organized collagen, and a dominant anti-inflammatory microenvironment. Laminar shear stress (LSS) maintains endothelial quiescence via KLF2/4. Resident M2-like macrophages (TREM2+) and Tregs, along with contractile SMCs, maintain structural integrity. EVs in this environment primarily carry atheroprotective cargoes (e.g., miR-146a). **(B)** Vulnerable Plaque (TCFA): Characterized by a thin cap (<65 μm), large necrotic core, and spatial disorganization. Oscillatory shear stress (OSS) and stiffness mismatch (due to microcalcifications) at the shoulder region trigger a pro-inflammatory cascade. **(C)** The Vicious Cycle of Instability: In the vulnerable shoulder, a self-amplifying loop is established: (1) Mechanical stress recruits M1 macrophages and CD8^+^ T cells via OSS-induced chemokines; (2) These cells secrete MMPs and pro-inflammatory EVs (miR-155), leading to collagen degradation and SMC apoptosis; (3) Structural weakening exacerbates stress concentration, further accelerating immune cell infiltration and ECM breakdown. **(D)** Rupture and Thrombosis: The culmination of this spatial disorganization is the physical failure of the fibrous cap, exposing the thrombogenic core and leading to acute clinical events.

Figure 5 illustrates the spatial determinants underlying the transition from stable to vulnerable atherosclerotic plaque and the events leading to rupture. Panel A shows the stable plaque architecture, in which LSS-maintained endothelial quiescence (KLF2/4), resident TREM2^+^ M2-like macrophages, Tregs, and contractile SMCs together maintain structural integrity, supported by atheroprotective EV cargoes (miR-146a). Panel B contrasts this with the thin-cap fibroatheroma (TCFA), where OSS and stiffness mismatch from microcalcifications at the shoulder initiate a pro-inflammatory cascade amid spatial disorganisation. Panel C delineates the instability loop in which mechanical stress recruits M1 macrophages and CD8^+^ T cells, whose MMP secretion and pro-inflammatory EVs (miR-155) drive collagen degradation and SMC apoptosis, further concentrating mechanical stress and accelerating ECM breakdown—each step feeding back to the upstream defect. Panel D shows the end point of spatial disorganisation: fibrous cap rupture, thrombogenic core exposure, and acute clinical events.

## 7 Research frontiers, translational significance, and future perspectives

### 7.1 Emerging technologies driving mechanistic insights

#### 7.1.1 Spatial transcriptomics elucidates the spatial heterogeneity of plaque immune microenvironments

Spatial transcriptomics has changed how atherosclerotic plaque heterogeneity can be studied. Platforms such as 10x Visium localise gene expression at high resolution while preserving tissue morphology, so that molecular signals can be mapped to defined plaque regions. Integration of scRNA-seq with spatial transcriptomics permits systematic reconstruction of plaque cellular composition and intercellular communication within its spatial architecture. Integrative analysis of 12 human atherosclerotic plaques at different disease stages identified a previously unappreciated role for the microvasculature in plaque progression and mapped lymphocyte recruitment pathways mediated by ACKR1-positive endothelial cells. Joint single-cell transcriptomic and chromatin accessibility profiling of human carotid plaques resolved 14 distinct cell populations with unique molecular signatures and activation states, including multiple pro-inflammatory macrophage subpopulations and endothelial cell subsets undergoing endothelial-to-mesenchymal transition—providing a high-resolution cellular atlas of the plaque immune microenvironment (Depuydt et al., 2020). Integration with multi-omics platforms encompassing proteomics and epigenomics is the next step (Rao et al., 2021).

##### 7.1.1.1 Validation utility for the MVDA model

Within the MVDA framework, spatial transcriptomics directly enables validation of forward coupling (C3) by co-localising mechanosensitive transcription signatures (KLF2, CTGF, CYR61) with hemodynamically defined plaque microregions, and of reverse coupling (C4) by identifying recipient-cell transcriptional signatures that match donor mechanical states—the latter being the cardinal experimental signature that would graduate C4 from [I] to [E].

#### 7.1.2 Advances in single-cell mechanical measurement

Single-cell mechanical measurement provides tools for *in situ* quantification of mechanical signals within the plaque microenvironment. Atomic force microscopy (AFM) nanoindentation measures elastic modulus, viscoelasticity, and local stiffness in living cells and tissues at nanometer spatial resolution, making it one of the most sensitive techniques available for tissue mechanical characterisation. AFM has been applied to mechanobiological profiling of cardiovascular and other complex human tissues, revealing spatial stiffness gradients and their relation to cellular mechanical states (Cho et al., 2023). Micropipette aspiration quantifies the deformability and cortical tension of individual plaque-infiltrating cells such as macrophages and endothelial cells, linking cellular mechanical properties to activation state and EV release behaviour. Current limitations include the absence of real-time *in vivo* measurement, the risk of altering native tissue mechanical properties during sample preparation, and the low throughput of high-resolution mechanical mapping. Integration of AFM with fluorescence imaging and spatial omics offers a route to joint high-resolution characterisation of mechanical and molecular features within plaque tissue (Magazzù and Marcuello, 2023).

##### 7.1.2.1 Validation utility for the MVDA model

AFM-based nanoindentation provides the quantitative mechanical ground truth required to anchor the MVDA model’s mechanical-stimulus axis (C1). Coupled with single-cell EV profiling on the same tissue section, it converts the model from *descriptive* to *dose-response testable*—that is, it allows the question “does a defined increment in matrix stiffness produce a defined increment in EV cargo composition?” to be addressed empirically rather than inferentially.

#### 7.1.3 *In vivo* vesicle tracking: Applications and challenges

Real-time *in vivo* tracking of EVs is a prerequisite for understanding their spatial distribution and targeted uptake within plaques. Nanoparticle tracking analysis (NTA) is in routine use for *in vitro* quantitative characterisation of EVs; combined with fluorescent labeling, it permits dynamic tracking of EV secretion, diffusion, and uptake in cell culture models. The MISEV2023 guidelines provide an internationally standardised framework for EV quality control (Welsh et al., 2024). A biomimetic delivery system based on engineered M2 macrophage-derived EVs fused with platelet membranes has been validated by fluorescence imaging to achieve targeted accumulation of EVs within plaques in ApoE^−/−^ mice (Xie et al., 2024). Current *in vivo* EV tracking faces several challenges: non-specific signals from free fluorescent dye limit labeling specificity; EVs are cleared rapidly in the circulation with short half-lives (minutes to hours); and high-resolution *in vivo* imaging methods that distinguish EVs of different cellular origins are not yet available. Tissue-specific targeting EV probes integrated with spatial omics offer a path forward.

##### 7.1.3.1 Validation utility for the MVDA model

*In vivo* EV tracking is the rate-limiting technology for empirical validation of the MVDA model’s reverse coupling. Until donor-recipient EV trafficking can be resolved spatiotemporally *in vivo*—with cell-of-origin identification, recipient cell-type identification, and cargo tracking on the same intact tissue—C4 remains supported principally by inference. This is the single most important methodological dependency of the framework, and is the reason that Section 6.4 stratifies C4-related claims at the [I] level.

### 7.2 Therapeutic strategies targeting mechanotransduction

#### 7.2.1 Piezo1 antagonists

The role of Piezo1 in mediating OSS-induced endothelial inflammation has identified it as a therapeutic target for atherosclerosis. The pharmacological antagonist GsMTx4, a peptide toxin derived from tarantula venom, reversibly inhibits Piezo1 channel opening by modifying membrane tension. In ApoE^−/−^ mice, GsMTx4 attenuated high-fat-diet-induced aortic root plaque burden and suppressed disturbed flow-mediated carotid plaque progression, with the responsible mechanisms including inhibition of the Ca^2+^/CaM/CaMKII/FAK/Src pathway and reduced YAP nuclear translocation (Lan et al., 2024) [P]. The principal limitations of GsMTx4 are poor *in vivo* stability and low oral bioavailability of peptide-based drugs, potential off-target effects given Piezo1 expression across multiple cell types, and the absence of long-term safety data. Piezo1-specific modulators and cell-type-selective genetic intervention strategies are under investigation (Tang H. et al., 2022).

#### 7.2.2 YAP/TAZ inhibitors

Verteporfin (VP), a benzoporphyrin derivative initially developed as a photosensitizer for photodynamic therapy, inhibits the YAP–TEAD transcriptional complex without photoactivation. A biomimetic targeted delivery system using monocyte membrane-coated nanoparticles (MoNPs) loaded with VP achieves selective delivery to activated endothelium lining atherosclerosis-prone arterial regions and suppresses plaque progression in ApoE^−/−^ mice (Huang et al., 2024). The strategy uses the inflammatory endothelial tropism of monocyte surface molecules to achieve lesion-site-selective YAP/TAZ inhibition, reducing off-target toxicity associated with systemic suppression. Translational challenges include the photosensitivity of VP (limiting systemic administration), the role of YAP/TAZ in development and tissue homeostasis (leaving the safety window for chronic inhibition undefined), and the need to establish clinical-grade manufacturing processes for plaque-specific delivery vehicles [P].

#### 7.2.3 Hemodynamic intervention strategies

Improving local arterial hemodynamics and reducing OSS exposure is a mechanotransduction-axis intervention strategy. The relationship between hemodynamics and endothelial function has been clarified in basic research, indicating that local FSS optimisation may attenuate endothelial inflammatory activation (Wang et al., 2023). Large-scale randomised controlled trials specifically targeting OSS reduction are, however, lacking, and the complete translational chain from hemodynamic improvement to immune microenvironment and plaque structural remodeling awaits systematic clinical evidence [I].

##### 7.2.3.1 Translational risk appraisal—mechanotransduction interventions

*Specificity*. Piezo1 and YAP/TAZ are broadly expressed across multiple cell types and tissues; current inhibitors (GsMTx4, verteporfin) lack tissue selectivity. Cell-membrane-coated biomimetic nanoparticle delivery [MoNP-VP (Huang et al., 2024); M2-EV–platelet membrane fusion (Xie et al., 2024)] improves plaque tropism in murine models but is unvalidated in large animals.

*Safety*. Chronic systemic YAP inhibition carries the risk of impairing tissue regeneration, given the role of YAP/TAZ in development and homeostasis. Chronic Piezo1 blockade affects red-cell volume regulation and lymphatic function; long-term safety data are absent. Hemodynamic intervention (Section 7.2.3) is the lowest-risk modality on this dimension but is anatomically constrained and is unlikely to be feasible for most coronary lesions.

### 7.3 Therapeutic potential of engineered extracellular vesicles

#### 7.3.1 Biomimetic EV strategies loaded with anti-inflammatory cargo

Engineered EVs combine the biocompatibility, low immunogenicity, and plaque-homing properties of natural EVs with artificial drug-loading capacity. Bone marrow-derived M2 macrophage exosomes (BMDM-IL-4-exo) carry anti-inflammatory miRNAs (miR-99a, miR-146b, miR-378a) that target NF-κB and TNF-α signaling, reducing excessive hematopoiesis and plaque inflammation and stabilising plaque in ApoE^−/−^ mice (Bouchareychas et al., 2020). A biomimetic delivery system based on engineered M2 macrophage-derived EVs fused with platelet membranes combines the inflammatory tropism of M2-type EVs with platelet-membrane-mediated plaque targeting, achieving efficient targeted accumulation and inflammatory resolution in ApoE^−/−^ mice (Xie et al., 2024). More broadly, cell-hitchhiking strategies that repurpose circulating neutrophils, platelets, macrophages, and red blood cells as programmable carriers exploit the same pathophysiologic triggers—oxidative stress, MMP activity, acidic pH, and shear stress—to achieve lesion-specific payload release (Heydari et al., 2026), a design logic that directly operationalises the mechanical and vesicular cues central to the MVDA model. For the MVDA framework, an interesting future direction is loading mechanosensory-antagonising miRNAs (anti-miR-92a) onto engineered EVs, exploiting their natural homing to OSS-exposed endothelium at plaque shoulder regions for intervention at mechanically activated signaling nodes [P].

#### 7.3.2 Preclinical data and translational challenges

Preclinical studies of engineered EVs for atherosclerosis treatment are predominantly conducted in ApoE^−/−^ and LDLR^−/−^ mouse models, which differ from human plaques in lipoprotein metabolism, biomechanical properties, plaque composition, and lesion progression rate, limiting direct extrapolation of preclinical findings. Additional translational challenges include: low production efficiency and high batch-to-batch variability of natural EVs; short circulation half-lives that limit targeting efficiency; cargo heterogeneity that requires adherence to MISEV2023 quality control standards (Welsh et al., 2024); and the need for systematic evaluation of the immunogenicity of engineered modifications. Multiple clinical trials of mesenchymal stem cell-derived EVs for inflammatory diseases are underway and provide regulatory precedents for EV clinical translation (Saleem et al., 2024).

##### 7.3.2.1 Translational risk appraisal—engineered EV platforms

*Scalability*. Engineered EV manufacturing remains low-throughput with high batch-to-batch variability; GMP-grade EV production is technically demanding and economically unproven, and the unit economics of plaque-targeted EV therapy at scale are uncertain. Compliance with MISEV2023 reporting standards (Welsh et al., 2024) is essential to support cross-study reproducibility and regulatory acceptance, but adds regulatory complexity at every stage of development. These scalability constraints, taken together with the specificity and safety considerations in Section 7.2, justify the readiness stratification summarised in Table 4.

**TABLE 4:** Translational Readiness Matrix for MVDA-targeted interventions.

Intervention	Mechanism	Translational readiness	Key barriers	Preclinical evidence
Statins + lifestyle (control arm)	Systemic (lipid lowering)	Near-term (deployed)	None (standard of care)	[E] Human RCTs
IL-1β blockade (canakinumab analogs)	Downstream inflammation	Near-term	Cost; infection risk	[E] CANTOS RCT (Ridker et al., 2017)
MoNP-verteporfin (YAP/TAZ)	Mechano-transcriptional	Mid-term	Photosensitivity; chronic YAP-loss safety window undefined	[P] ApoE^−/−^ (Huang et al., 2024)
GsMTx4/Piezo1 antagonists	Mechano-sensor	Mid-term	Peptide PK; off-target across Piezo1-expressing tissues	[P] ApoE^−/−^ (Lan et al., 2024)
Engineered M2-EVs (anti-miR-92a)	Vesicular axis (C2/C4)	Mid-to-long-term	GMP scale-up; biodistribution; batch variability	[P] ApoE^−/−^ (Bouchareychas et al., 2020; Xie et al., 2024)
Circulating EV-miRNA panel (Dx)	Biomarker	Mid-term	Specificity; cross-platform standardisation	[I] cohort (Chang et al., 2019; Brandes et al., 2024)
Hemodynamic optimisation	Upstream mechanics (C1)	Long-term	Anatomic feasibility; lack of RCT evidence	[I] modeling

Evidence tiers ([E], [P], [I]) are defined in Table 2. EV, extracellular vesicle; GMP, good manufacturing practice; MVDA, mechano-vesicular dual-axis; PK, pharmacokinetics; RCT, randomised controlled trial. Numbers in parentheses refer to references in the reference list.

### 7.4 Biomarker development

#### 7.4.1 Circulating EV-derived miRNAs as non-invasive predictors of plaque vulnerability

Circulating EV-encapsulated miRNAs are protected from RNase degradation by the lipid bilayer, are reproducibly measurable, and are amenable to non-invasive liquid biopsy. miR-92a is secreted in vesicular form by endothelial cells under atheroprone conditions and participates in endothelial cell–macrophage intercellular signaling by suppressing KLF4 expression to support plaque progression; its elevated levels in plasma of coronary artery disease patients support its candidacy as a circulating biomarker of plaque disease state (Chang et al., 2019) [P]. microRNA sequencing of circulating EVs from patients with carotid artery stenosis has identified upregulation of miR-193b-5p, miR-193a-5p, and miR-125a-3p in patient serum, with concordant expression in corresponding plaque tissue, indicating that circulating EVs can reflect local plaque pathology (Brandes et al., 2024) [I]. Circulating EV-miRNA profiles (including miR-92a-3p and related species) differ between atherosclerosis patients and healthy controls, supporting their use as dynamic indicators of disease state (Patel et al., 2023).

Specificity is the principal barrier to clinical deployment of circulating EV-miRNA panels: miR-92a, miR-155, and related species are co-elevated across multiple inflammatory cardiovascular and metabolic conditions, undermining single-marker discrimination of plaque vulnerability. Two strategies may address this. (i) Multi-marker composite scoring—combining mechanosensitive (miR-92a), inflammatory (miR-155), and efferocytosis-related (MerTK-shed soluble protein) signals into a single panel that reflects multiple MVDA model components simultaneously. (ii) Multimodal integration—coupling liquid-biopsy EV signatures with FDG-PET for inflammation, 18F-NaF PET for microcalcification, and OCT for fibrous cap thickness, anchored to spatial transcriptomic reference atlases. Such an integrated diagnostic framework—rather than any single biomarker—is the realistic translational path. Prospective large-cohort studies validating independent predictive value for cardiovascular events remain the principal evidentiary gap.

### 7.5 Current challenges and future perspectives

At the methodological level, real-time *in situ* quantification of mechanical signals within plaques remains a bottleneck. Current AFM-based tools are constrained by invasiveness and limited temporal resolution for *in vivo* plaque measurements, and high-resolution *in vivo* EV tracking has no mature solution.

At the causal validation level, the bidirectional regulatory relationship between mechanotransduction and vesicular trafficking is at present supported principally by correlational evidence and *in vitro* intervention experiments. Direct genetic validation—through endothelial-cell-specific conditional Rab27a/b knockout or mechanoreceptor-specific knockout combined with *in vivo* vesicle tracing—is the principal outstanding requirement for graduating the MVDA reverse-coupling claim (Box 2, C4) from [I] to [E].

At the translational level, the gap between animal models and human plaque is the most prominent challenge. Murine atherosclerosis models differ from human plaques in lipoprotein metabolism, mechanical properties, plaque composition, and lesion progression rate. Future work should validate the MVDA dual-axis mechanism in large-animal models (porcine, non-human primate) combined with spatial omics analysis of human *ex vivo* plaques to establish cross-species validation (Sopic et al., 2023).

At the data integration level, joint multi-omics analysis integrating spatial transcriptomics, proteomics, and lipidomics is the methodological direction for systematic understanding of plaque mechano-immune interactions. Computational frameworks and standardisation pipelines for cross-omics data integration remain immature, and the trade-off between cellular resolution and spatial plaque coverage requires further optimisation (Vandereyken et al., 2023).

Three directions of future work are particularly likely to advance translation: construction of plaque “mechano-transcriptomic” atlases through integrated spatial multi-omics and single-cell mechanical measurement; prospective clinical cohort validation of mechanosensory-responsive EV biomarker panels with adequate specificity for mechanically activated states; and precision targeted intervention at key mechanotransduction nodes (Piezo1, YAP/TAZ) using biomimetic engineered EVs (Olejarz et al., 2023; Han et al., 2025).

#### 7.5.1 Clinical translation requirements

Advancing any MVDA-targeted intervention beyond preclinical phase requires, at minimum: (i) biodistribution and pharmacokinetics in large animals (porcine atherosclerosis model with human-like lipid metabolism); (ii) hemocompatibility testing per ISO 10993-4 for nanoparticle and EV-based platforms; (iii) off-target immune activation assessment (complement activation, cytokine storm risk); (iv) imaging-guided efficacy readouts—FDG-PET for inflammation, OCT for cap-thickness change, 18F-NaF PET for microcalcification—to support endpoint definition in early-phase trials; and (v) MISEV2023-compliant characterisation (Welsh et al., 2024) for all EV-based candidates. These five requirements operationalise the readiness tiers shown in Table 4.

## 8 Conclusion

Atherosclerotic plaque formation and progression is a process of multi-cellular and multi-signal dysregulation that unfolds within a defined spatial architecture. The intraplaque immune microenvironment is not homogeneous: it is spatially heterogeneous, shaped by hemodynamic conditions, extracellular matrix biomechanical properties, and EV-mediated intercellular communication. Defining the molecular basis of plaque spatial architecture is a prerequisite for developing precision intervention strategies.

The OSS-exposed shoulder regions, the foam-cell-dense zone surrounding the lipid necrotic core, and the structural integrity of the fibrous cap together constitute the spatial determinants of plaque vulnerability. Expansion of the lipid necrotic core and the outward spread of necro-inflammatory signals are propagated toward the fibrous cap through vesicle-mediated mechanisms. This spatially organised signal transmission pattern drives the transition from stable to vulnerable plaque phenotype and defines the principal target zones for spatially precise intervention.

We formally propose the Mechano-Vesicular Dual-Axis (MVDA) model, which posits that mechanotransduction and vesicular trafficking do not operate as independent regulatory pathways but form a deeply interconnected network through shared signaling nodes. Along the mechanotransduction axis, oscillatory shear stress and matrix stiffness gradients activate the RhoA/ROCK, PI3K/Akt, YAP/TAZ, and NF-κB cascades in endothelial cells and macrophages through mechanosensors including Piezo1 and integrins, organising the spatial distribution of pro-inflammatory gene programmes. Along the vesicular trafficking axis, mechanically activated cells secrete EVs in large quantity that carry pro-inflammatory miRNAs, oxidised lipids, and damage-associated molecular patterns, allowing mechanically activated immune signals to reach distal cells beyond direct paracrine range. The interconnection between the two axes is bidirectional: mechanical signals regulate vesicle biogenesis and cargo composition through multivesicular body fate, Rab GTPase subcellular localisation, and ESCRT pathway activity; vesicles carrying mechanosensory molecules in turn reconstitute mechanically activated signaling states in recipient cells, modulating their sensitivity to subsequent mechanical stimuli—the “spatial permeation” effect introduced in Section 5.3. KLF2/4, YAP/TAZ, NF-κB, PI3K/Akt, and mTOR are the shared signaling nodes that connect both axes, integrating mechanical inputs with vesicular outputs to set the spatial order of local immune homeostasis within the plaque.

Grounded in the MVDA model, the central paradigm shift in future plaque-stabilisation therapy is the move from systemic lipid-lowering or anti-inflammatory intervention toward spatially precise targeting of mechanically high-risk plaque microregions. Three mutually reinforcing directions support this shift: construction of plaque “mechano-transcriptomic” atlases through integration of spatial transcriptomics and single-cell mechanical measurement, to provide a spatial data foundation for target selection; development of engineered EV delivery systems that home to high-mechanical-activity plaque regions and deliver Piezo1 antagonists, YAP/TAZ inhibitors, or anti-inflammatory miRNAs to locally dysregulated microenvironments; and establishment of standardised detection protocols for mechanosensory miRNAs (such as miR-92a and miR-21) in circulating EVs, to construct liquid biopsy biomarker panels reflecting plaque mechanical activation states and vulnerability.

### Central contribution

This review establishes the Mechano-Vesicular Dual-Axis (MVDA) model as a unifying paradigm for atherosclerotic plaque immunobiology. The MVDA model reframes plaque vulnerability *not* as a localised inflammatory failure, but as the collapse of coherent coupling between mechanical and vesicular regulatory axes. As stratified by Box 1 and confirmed in Section 6.4, the mechano-immune scaffold (C1, C5) is already substantially human-validated; the vesicular reverse-coupling claims (C2–C4) remain forward-looking hypotheses awaiting *in vivo* genetic validation. We anticipate that the MVDA framework will catalyse a shift from systemic to spatially-precise diagnostic and therapeutic strategies, defining a new conceptual baseline for vulnerable-plaque medicine—and identifying, in C4, the single most consequential experimental question for the field to resolve in the coming decade.

## Glossary

ADAM17: A Disintegrin And Metalloproteinase 17
ALP: Alkaline Phosphatase
AMPK: AMP-activated Protein Kinase
CaMKII: Calcium/Calmodulin-dependent Protein Kinase II
CD47: Cluster of Differentiation 47
CaMKII: Calcium/Calmodulin-dependent Protein Kinase II
DAMP: Damage-Associated Molecular Pattern
ECM: Extracellular Matrix
ERK5: Extracellular signal-Regulated Kinase 5
EC: Endothelial Cell
EV: Extracellular Vesicle
FAK: Focal Adhesion Kinase
HIF-1α: Hypoxia-Inducible Factor 1-alpha
HMGB1: High Mobility Group Box 1
HSP: Heat Shock Protein
KLF2/4: Krüppel-Like Factor 2/4
LRNC: Lipid-Rich Necrotic Core
LSS: Laminar Shear Stress
MerTK: Mer Tyrosine Kinase
MHC-II: Major Histocompatibility Complex II
miR-21: microRNA-21
miR-146a: microRNA-146a
miR-155: microRNA-155
MMP: Matrix Metalloproteinase
mTOR: Mechanistic Target of Rapamycin
MVB: Multivesicular Body
NF-κB: Nuclear Factor kappa B
NLRP3: NLR Family Pyrin Domain-containing Protein 3
OSS: Oscillatory Shear Stress
PI3K: Phosphoinositide 3-Kinase
Piezo1: Piezo-type Mechanosensitive Ion Channel Component 1
PS: Phosphatidylserine
RhoA/ROCK: Ras Homolog A/Rho-associated Coiled-coil-containing Kinase
ROS: Reactive Oxygen Species
Runx2: Runt-related Transcription Factor 2
SMC: Smooth Muscle Cell
SPP1: Secreted Phosphoprotein 1
TCFA: Thin-Cap Fibroatheroma
TAZ: Transcriptional Co-activator with PDZ-binding motif
Th1: T helper 1 cell
TREM2: Triggering Receptor Expressed on Myeloid cells 2
Treg: Regulatory T cell
VSMC: Vascular Smooth Muscle Cell
WSS: Wall Shear Stress
YAP: Yes-Associated Protein

## References

[B1] Alencar G. F. Owsiany K. M. Karnewar S. Sukhavasi K. Mocci G. Nguyen A. T. (2020). Stem cell pluripotency genes Klf4 and Oct4 regulate complex SMC phenotypic changes critical in late-stage atherosclerotic lesion pathogenesis. Circulation 142 (21), 2045–2059. 10.1161/circulationaha.120.046672 32674599 PMC7682794

[B2] Alonso-Herranz L. Albarrán-Juárez J. Bentzon J. F. (2023). Mechanisms of fibrous cap formation in atherosclerosis. Front. Cardiovasc Med. 10, 1254114. 10.3389/fcvm.2023.1254114 37671141 PMC10475556

[B3] Atcha H. Jairaman A. Holt J. R. Meli V. S. Nagalla R. R. Veerasubramanian P. K. (2021). Mechanically activated ion channel Piezo1 modulates macrophage polarization and stiffness sensing. Nat. Commun. 12 (1), 3256. 10.1038/s41467-021-23482-5 34059671 PMC8167181

[B4] Atcha H. Kulkarni D. Meli V. S. Veerasubramanian P. K. Wang Y. Cahalan M. D. (2024). Piezo1-mediated mechanotransduction enhances macrophage oxidized low-density lipoprotein uptake and atherogenesis. PNAS Nexus 3 (11), pgae436. 10.1093/pnasnexus/pgae436 39544498 PMC11563038

[B5] Bäck M. Yurdagul A. Jr. Tabas I. Öörni K. Kovanen P. T. (2019). Inflammation and its resolution in atherosclerosis: mediators and therapeutic opportunities. Nat. Rev. Cardiol. 16 (7), 389–406. 10.1038/s41569-019-0169-2 30846875 PMC6727648

[B6] Bentzon J. F. Otsuka F. Virmani R. Falk E. (2014). Mechanisms of plaque formation and rupture. Circ. Res. 114 (12), 1852–1866. 10.1161/circresaha.114.302721 24902970

[B7] Bouchareychas L. Duong P. Covarrubias S. Alsop E. Phu T. A. Chung A. (2020). Macrophage exosomes resolve atherosclerosis by regulating hematopoiesis and inflammation via MicroRNA cargo. Cell Rep. 32 (2), 107881. 10.1016/j.celrep.2020.107881 32668250 PMC8143919

[B8] Brandes F. Meidert A. S. Kirchner B. Yu M. Gebhardt S. Steinlein O. K. (2024). Identification of microRNA biomarkers simultaneously expressed in circulating extracellular vesicles and atherosclerotic plaques. Front. Cardiovasc Med. 11, 1307832. 10.3389/fcvm.2024.1307832 38725837 PMC11079260

[B9] Buzas E. I. (2023). The roles of extracellular vesicles in the immune system. Nat. Rev. Immunol. 23 (4), 236–250. 10.1038/s41577-022-00763-8 35927511 PMC9361922

[B10] Cai B. Thorp E. B. Doran A. C. Sansbury B. E. Daemen M. J. Dorweiler B. (2017). MerTK receptor cleavage promotes plaque necrosis and defective resolution in atherosclerosis. J. Clin. Invest. 127 (2), 564–568. 10.1172/jci90520 28067670 PMC5272169

[B11] Chang Y. J. Li Y. S. Wu C. C. Wang K. C. Huang T. C. Chen Z. (2019). Extracellular MicroRNA-92a mediates endothelial cell-macrophage communication. Arterioscler. Thromb. Vasc. Biol. 39 (12), 2492–2504. 10.1161/atvbaha.119.312707 31597449 PMC6879873

[B12] Chatzizisis Y. S. Coskun A. U. Jonas M. Edelman E. R. Feldman C. L. Stone P. H. (2007). Role of endothelial shear stress in the natural history of coronary atherosclerosis and vascular remodeling: molecular, cellular, and vascular behavior. J. Am. Coll. Cardiol. 49 (25), 2379–2393. 10.1016/j.jacc.2007.02.059 17599600

[B13] Cheng H. Zhong W. Wang L. Zhang Q. Ma X. Wang Y. (2023). Effects of shear stress on vascular endothelial functions in atherosclerosis and potential therapeutic approaches. Biomed. Pharmacother. 158, 114198. 10.1016/j.biopha.2022.114198 36916427

[B14] Chiu J. J. Chien S. (2011). Effects of disturbed flow on vascular endothelium: pathophysiological basis and clinical perspectives. Physiol. Rev. 91 (1), 327–387. 10.1152/physrev.00047.2009 21248169 PMC3844671

[B15] Cho D. H. Aguayo S. Cartagena-Rivera A. X. (2023). Atomic force microscopy-mediated mechanobiological profiling of complex human tissues. Biomaterials 303, 122389. 10.1016/j.biomaterials.2023.122389 37988897 PMC10842832

[B16] Chu T. Xu X. Ruan Z. Wu L. Zhou M. Zhu G. (2022). miR-146a contributes to atherosclerotic plaque stability by regulating the expression of TRAF6 and IRAK-1. Mol. Biol. Rep. 49 (6), 4205–4216. 10.1007/s11033-022-07253-z 35195809

[B17] Chung J. Kim K. H. Yu N. An S. H. Lee S. Kwon K. (2022). Fluid shear stress regulates the landscape of microRNAs in endothelial cell-derived small extracellular vesicles and modulates the function of endothelial cells. Int. J. Mol. Sci. 23 (3), 1314. 10.3390/ijms23031314 35163238 PMC8836123

[B18] Coly P. M. Chatterjee S. Mezine F. Jekmek C. E. Devue C. Nipoti T. (2024). Low fluid shear stress stimulates the uptake of noxious endothelial extracellular vesicles via MCAM and PECAM-1 cell adhesion molecules. J. Extracell. Vesicles 13 (10), e12414. 10.1002/jev2.12414 39400522 PMC11472237

[B19] Corti A. De Paolis A. Grossman P. Dinh P. A. Aikawa E. Weinbaum S. (2022). The effect of plaque morphology, material composition and microcalcifications on the risk of cap rupture: a structural analysis of vulnerable atherosclerotic plaques. Front. Cardiovasc Med. 9, 1019917. 10.3389/fcvm.2022.1019917 36277774 PMC9583261

[B20] Davidson S. M. Boulanger C. M. Aikawa E. Badimon L. Barile L. Binder C. J. (2023). Methods for the identification and characterization of extracellular vesicles in cardiovascular studies: from exosomes to microvesicles. Cardiovasc Res. 119 (1), 45–63. 10.1093/cvr/cvac031 35325061 PMC10233250

[B21] Davis M. J. Earley S. Li Y. S. Chien S. (2023). Vascular mechanotransduction. Physiol. Rev. 103 (2), 1247–1421. 10.1152/physrev.00053.2021 36603156 PMC9942936

[B22] Deng W. Tang T. Hou Y. Zeng Q. Wang Y. Fan W. (2019). Extracellular vesicles in atherosclerosis. Clin. Chim. Acta 495, 109–117. 10.1016/j.cca.2019.04.051 30959044

[B23] Depuydt M. A. C. Prange K. H. M. Slenders L. Örd T. Elbersen D. Boltjes A. (2020). Microanatomy of the human atherosclerotic plaque by single-cell transcriptomics. Circ. Res. 127 (11), 1437–1455. 10.1161/circresaha.120.316770 32981416 PMC7641189

[B24] Di X. Gao X. Peng L. Ai J. Jin X. Qi S. (2023). Cellular mechanotransduction in health and diseases: from molecular mechanism to therapeutic targets. Signal Transduct. Target Ther. 8 (1), 282. 10.1038/s41392-023-01501-9 37518181 PMC10387486

[B25] Doran A. C. Yurdagul A. Jr. Tabas I. (2020). Efferocytosis in health and disease. Nat. Rev. Immunol. 20 (4), 254–267. 10.1038/s41577-019-0240-6 31822793 PMC7667664

[B26] Duewell P. Kono H. Rayner K. J. Sirois C. M. Vladimer G. Bauernfeind F. G. (2010). NLRP3 inflammasomes are required for atherogenesis and activated by cholesterol crystals. Nature 464 (7293), 1357–1361. 10.1038/nature08938 20428172 PMC2946640

[B27] Elmarasi M. Elmakaty I. Elsayed B. Elsayed A. Zein J. A. Boudaka A. (2024). Phenotypic switching of vascular smooth muscle cells in atherosclerosis, hypertension, and aortic dissection. J. Cell Physiol. 239 (4), e31200. 10.1002/jcp.31200 38291732

[B28] Emoto T. Yamamoto H. Yamashita T. Takaya T. Sawada T. Takeda S. (2022). Single-cell RNA sequencing reveals a distinct immune landscape of myeloid cells in coronary culprit plaques causing acute coronary syndrome. Circulation 145 (18), 1434–1436. 10.1161/circulationaha.121.058414 35500048

[B29] Falk E. Nakano M. Bentzon J. F. Finn A. V. Virmani R. (2013). Update on acute coronary syndromes: the pathologists' view. Eur. Heart J. 34 (10), 719–728. 10.1093/eurheartj/ehs411 23242196

[B30] Fernandez D. M. Rahman A. H. Fernandez N. F. Chudnovskiy A. Amir E. D. Amadori L. (2019). Single-cell immune landscape of human atherosclerotic plaques. Nat. Med. 25 (10), 1576–1588. 10.1038/s41591-019-0590-4 31591603 PMC7318784

[B31] Fidler T. P. Xue C. Yalcinkaya M. Hardaway B. Abramowicz S. Xiao T. (2021). The AIM2 inflammasome exacerbates atherosclerosis in clonal haematopoiesis. Nature 592 (7853), 296–301. 10.1038/s41586-021-03341-5 33731931 PMC8038646

[B32] Gimbrone M. A. Jr. García-Cardeña G. (2016). Endothelial cell dysfunction and the pathobiology of atherosclerosis. Circ. Res. 118 (4), 620–636. 10.1161/circresaha.115.306301 26892962 PMC4762052

[B33] Givens C. Tzima E. (2016). Endothelial mechanosignaling: does one sensor fit all? Antioxid. Redox Signal 25 (7), 373–388. 10.1089/ars.2015.6493 27027326 PMC5011625

[B34] Gomez I. Ward B. Souilhol C. Recarti C. Ariaans M. Johnston J. (2020). Neutrophil microvesicles drive atherosclerosis by delivering miR-155 to atheroprone endothelium. Nat. Commun. 11 (1), 214. 10.1038/s41467-019-14043-y 31924781 PMC6954269

[B35] Grebe A. Hoss F. Latz E. (2018). NLRP3 inflammasome and the IL-1 pathway in atherosclerosis. Circ. Res. 122 (12), 1722–1740. 10.1161/circresaha.118.311362 29880500

[B36] Griendling K. K. Sorescu D. Ushio-Fukai M. (2000). NAD(P)H oxidase: role in cardiovascular biology and disease. Circ. Res. 86 (5), 494–501. 10.1161/01.res.86.5.494 10720409

[B37] Han Y. He M. Marin T. Shen H. Wang W. T. Lee T. Y. (2021). Roles of KLF4 and AMPK in the inhibition of glycolysis by pulsatile shear stress in endothelial cells. Proc. Natl. Acad. Sci. U. S. A. 118 (21), 118. 10.1073/pnas.2103982118 PMC8166073 34001623

[B38] Han Q. F. Li W. J. Hu K. S. Gao J. Zhai W. L. Yang J. H. (2022). Exosome biogenesis: machinery, regulation, and therapeutic implications in cancer. Mol. Cancer 21 (1), 207. 10.1186/s12943-022-01671-0 36320056 PMC9623991

[B39] Han Y. Xu H. Yao X. Li Z. Zhu C. Li X. (2025). Molecular mechanisms and therapeutic progress in atherosclerosis: bridging immune inflammation and precision medicine. Front. Immunol. 16, 1737662. 10.3389/fimmu.2025.1737662 41562048 PMC12812629

[B40] Hartman C. D. Isenberg B. C. Chua S. G. Wong J. Y. (2016). Vascular smooth muscle cell durotaxis depends on extracellular matrix composition. Proc. Natl. Acad. Sci. U. S. A. 113 (40), 11190–11195. 10.1073/pnas.1611324113 27647912 PMC5056055

[B41] He S. Wu C. Xiao J. Li D. Sun Z. Li M. (2018). Endothelial extracellular vesicles modulate the macrophage phenotype: potential implications in atherosclerosis. Scand. J. Immunol. 87 (4), e12648. 10.1111/sji.12648 29465752

[B42] He L. Zhang C. L. Chen Q. Wang L. Huang Y. (2022). Endothelial shear stress signal transduction and atherogenesis: from mechanisms to therapeutics. Pharmacol. Ther. 235, 108152. 10.1016/j.pharmthera.2022.108152 35122834

[B43] Heydari F. Hamblin M. R. Naghinezhad J. (2026). From inflammation to targeted therapy: leveraging neutrophil, platelet, RBC, and macrophage biology for nanocarrier in atherosclerosis. Curr. Atheroscler. Rep. 28 (1), 26. 10.1007/s11883-026-01394-3 41733760

[B44] Holzapfel G. A. Sommer G. Regitnig P. (2004). Anisotropic mechanical properties of tissue components in human atherosclerotic plaques. J. Biomech. Eng. 126 (5), 657–665. 10.1115/1.1800557 15648819

[B45] Hou Z. Deng L. Fang F. Zhao T. Zhang Y. Li G. (2025). Endothelial cells under disturbed flow release extracellular vesicles to promote inflammatory polarization of macrophages and accelerate atherosclerosis. BMC Biol. 23 (1), 20. 10.1186/s12915-025-02125-x 39838385 PMC11753076

[B46] Hu X. Nan Y. Zhang Y. Fan J. Wang H. Bai Y. (2024). Simultaneously blocking ANGPTL3 and CD47 prevents the progression of atherosclerosis through regulating lipid metabolism, macrophagic efferocytosis and lipid peroxidation. Pharmacol. Res. 210, 107486. 10.1016/j.phrs.2024.107486 39488258

[B47] Huang H. C. Wang T. Y. Rousseau J. Orlando M. Mungaray M. Michaud C. (2024). Biomimetic nanodrug targets inflammation and suppresses YAP/TAZ to ameliorate atherosclerosis. Biomaterials 306, 122505. 10.1016/j.biomaterials.2024.122505 38359507 PMC11479593

[B48] Iranpanah A. Kooshki L. Moradi S. Z. Saso L. Fakhri S. Khan H. (2023). The exosome-mediated PI3K/Akt/mTOR signaling pathway in neurological diseases. Pharmaceutics 15 (3), 1006. 10.3390/pharmaceutics15031006 36986865 PMC10057486

[B49] Jaitin D. A. Adlung L. Thaiss C. A. Weiner A. Li B. Descamps H. (2019). Lipid-associated macrophages control metabolic homeostasis in a Trem2-dependent manner. Cell 178 (3), 686–698.e614. 10.1016/j.cell.2019.05.054 31257031 PMC7068689

[B50] Jansen I. Cahalane R. Hengst R. Akyildiz A. Farrell E. Gijsen F. (2024). The interplay of collagen, macrophages, and microcalcification in atherosclerotic plaque cap rupture mechanics. Basic Res. Cardiol. 119 (2), 193–213. 10.1007/s00395-024-01033-5 38329498 PMC11008085

[B51] Jiang F. Zhang B. Zhang X. Zhang R. Lu Q. Shi F. (2023). miRNA-92a inhibits vascular smooth muscle cell phenotypic modulation and may help prevent in-stent restenosis. Mol. Med. Rep. 27 (2), 40. 10.3892/mmr.2023.12927 36601739 PMC9835053

[B52] Jinnouchi H. Sato Y. Sakamoto A. Cornelissen A. Mori M. Kawakami R. (2020). Calcium deposition within coronary atherosclerotic lesion: implications for plaque stability. Atherosclerosis 306, 85–95. 10.1016/j.atherosclerosis.2020.05.017 32654790

[B53] Johnson J. L. (2017). Metalloproteinases in atherosclerosis. Eur. J. Pharmacol. 816, 93–106. 10.1016/j.ejphar.2017.09.007 28893577

[B54] Joshi D. Coon B. G. Chakraborty R. Deng H. Yang Z. Babar M. U. (2024). Endothelial γ-protocadherins inhibit KLF2 and KLF4 to promote atherosclerosis. Nat. Cardiovasc Res. 3 (9), 1035–1048. 10.1038/s44161-024-00522-z 39232138 PMC11399086

[B55] Kapustin A. N. Chatrou M. L. Drozdov I. Zheng Y. Davidson S. M. Soong D. (2015). Vascular smooth muscle cell calcification is mediated by regulated exosome secretion. Circ. Res. 116 (8), 1312–1323. 10.1161/circresaha.116.305012 25711438

[B56] Kawtharany L. Bessueille L. Issa H. Hamade E. Zibara K. Magne D. (2022). Inflammation and microcalcification: a never-ending vicious cycle in atherosclerosis? J. Vasc. Res. 59 (3), 137–150. 10.1159/000521161 35038712

[B57] Kedhi E. Berta B. Roleder T. Hermanides R. S. Fabris E. Ajj I. J. (2021). Thin-cap fibroatheroma predicts clinical events in diabetic patients with normal fractional flow reserve: the COMBINE OCT-FFR trial. Eur. Heart J. 42 (45), 4671–4679. 10.1093/eurheartj/ehab433 34345911

[B58] Kong P. Cui Z. Y. Huang X. F. Zhang D. D. Guo R. J. Han M. (2022). Inflammation and atherosclerosis: signaling pathways and therapeutic intervention. Signal Transduct. Target Ther. 7 (1), 131. 10.1038/s41392-022-00955-7 35459215 PMC9033871

[B59] Kumar D. Pandit R. Yurdagul A. Jr. (2023). Mechanisms of continual efferocytosis by macrophages and its role in mitigating atherosclerosis. Immunometabolism (Cobham) 5 (1), e00017. 10.1097/in9.0000000000000017 36710920 PMC9869949

[B60] Kwak B. R. Bäck M. Bochaton-Piallat M. L. Caligiuri G. Daemen M. J. Davies P. F. (2014). Biomechanical factors in atherosclerosis: mechanisms and clinical implications. Eur. Heart J. 35 (43), 3013–3020. 10.1093/eurheartj/ehu353 25230814 PMC4810806

[B61] Kyaw T. Winship A. Tay C. Kanellakis P. Hosseini H. Cao A. (2013). Cytotoxic and proinflammatory CD8+ T lymphocytes promote development of vulnerable atherosclerotic plaques in apoE-deficient mice. Circulation 127 (9), 1028–1039. 10.1161/circulationaha.112.001347 23395974

[B62] Lan Y. Lu J. Zhang S. Jie C. Chen C. Xiao C. (2024). Piezo1-mediated mechanotransduction contributes to disturbed flow-induced atherosclerotic endothelial inflammation. J. Am. Heart Assoc. 13 (21), e035558. 10.1161/jaha.123.035558 39450718 PMC11935715

[B63] Li W. Huang Y. Liu J. Zhou Y. Sun H. Fan Y. (2024). Defective macrophage efferocytosis in advanced atherosclerotic plaque and mitochondrial therapy. Life Sci. 359, 123204. 10.1016/j.lfs.2024.123204 39491771

[B64] Li C. Fang F. Wang E. Yang H. Yang X. Wang Q. (2025). Engineering extracellular vesicles derived from endothelial cells sheared by laminar flow for anti-atherosclerotic therapy through reprogramming macrophage. Biomaterials 314, 122832. 10.1016/j.biomaterials.2024.122832 39270628

[B65] Libby P. (2021). The changing landscape of atherosclerosis. Nature 592 (7855), 524–533. 10.1038/s41586-021-03392-8 33883728

[B66] Libby P. Loscalzo J. Ridker P. M. Farkouh M. E. Hsue P. Y. Fuster V. (2018). Inflammation, immunity, and infection in atherothrombosis: JACC review topic of the week. J. Am. Coll. Cardiol. 72 (17), 2071–2081. 10.1016/j.jacc.2018.08.1043 30336831 PMC6196735

[B67] Libby P. Pasterkamp G. Crea F. Jang I. K. (2019). Reassessing the mechanisms of acute coronary syndromes. Circ. Res. 124 (1), 150–160. 10.1161/circresaha.118.311098 30605419 PMC6447371

[B68] Lim X. R. Harraz O. F. (2024). Mechanosensing by vascular endothelium. Annu. Rev. Physiol. 86, 71–97. 10.1146/annurev-physiol-042022-030946 37863105 PMC10922104

[B69] Liu C. S. C. Mandal T. Biswas P. Hoque M. A. Bandopadhyay P. Sinha B. P. (2024). Piezo1 mechanosensing regulates integrin-dependent chemotactic migration in human T cells. Elife 12, 12. 10.7554/eLife.91903 PMC10942591 38393325

[B70] Loyer X. Zlatanova I. Devue C. Yin M. Howangyin K. Y. Klaihmon P. (2018). Intra-cardiac release of extracellular vesicles shapes inflammation following myocardial infarction. Circ. Res. 123 (1), 100–106. 10.1161/circresaha.117.311326 29592957 PMC6023578

[B71] Ma Q. Fan Q. Han X. Dong Z. Xu J. Bai J. (2021). Platelet-derived extracellular vesicles to target plaque inflammation for effective anti-atherosclerotic therapy. J. Control Release 329, 445–453. 10.1016/j.jconrel.2020.11.064 33285103

[B72] Magazzù A. Marcuello C. (2023). Investigation of soft matter nanomechanics by atomic force microscopy and optical tweezers: a comprehensive review. Nanomaterials (Basel) 13 (6), 963. 10.3390/nano13060963 36985857 PMC10053849

[B73] Marsch E. Sluimer J. C. Daemen M. J. (2013). Hypoxia in atherosclerosis and inflammation. Curr. Opin. Lipidol. 24 (5), 393–400. 10.1097/MOL.0b013e32836484a4 23942270

[B74] Mei F. Guo Y. Wang Y. Zhou Y. Heng B. C. Xie M. (2024). Matrix stiffness regulates macrophage polarisation via the Piezo1-YAP signalling axis. Cell Prolif. 57 (8), e13640. 10.1111/cpr.13640 38556840 PMC11294424

[B75] Meli V. S. Atcha H. Veerasubramanian P. K. Nagalla R. R. Luu T. U. Chen E. Y. (2020). YAP-mediated mechanotransduction tunes the macrophage inflammatory response. Sci. Adv. 6 (49), eabb8471. 10.1126/sciadv.abb8471 33277245 PMC7717914

[B76] Mensah G. A. Fuster V. Murray C. J. L. Roth G. A. (2023). Global burden of cardiovascular diseases and risks, 1990-2022. J. Am. Coll. Cardiol. 82 (25), 2350–2473. 10.1016/j.jacc.2023.11.007 38092509 PMC7615984

[B77] Michel J. B. Virmani R. Arbustini E. Pasterkamp G. (2011). Intraplaque haemorrhages as the trigger of plaque vulnerability. Eur. Heart J. 32 (16), 1977–1985. 10.1093/eurheartj/ehr054 21398643 PMC3155759

[B78] Mittelheisser V. Gensbittel V. Bonati L. Li W. Tang L. Goetz J. G. (2024). Evidence and therapeutic implications of biomechanically regulated immunosurveillance in cancer and other diseases. Nat. Nanotechnol. 19 (3), 281–297. 10.1038/s41565-023-01535-8 38286876

[B79] Montanaro M. Scimeca M. Anemona L. Servadei F. Giacobbi E. Bonfiglio R. (2021). The paradox effect of calcification in carotid atherosclerosis: microcalcification is correlated with plaque instability. Int. J. Mol. Sci. 22 (1), 395. 10.3390/ijms22010395 33401449 PMC7796057

[B80] Mosquera J. V. Auguste G. Wong D. Turner A. W. Hodonsky C. J. Alvarez-Yela A. C. (2023). Integrative single-cell meta-analysis reveals disease-relevant vascular cell states and markers in human atherosclerosis. Cell Rep. 42 (11), 113380. 10.1016/j.celrep.2023.113380 37950869 PMC12335892

[B81] Mukhopadhyay A. Tsukasaki Y. Chan W. C. Le J. P. Kwok M. L. Zhou J. (2024). trans-Endothelial neutrophil migration activates bactericidal function via Piezo1 mechanosensing. Immunity 57 (1), 52–67.e10. 10.1016/j.immuni.2023.11.007 38091995 PMC10872880

[B82] Multhaupt H. A. Leitinger B. Gullberg D. Couchman J. R. (2016). Extracellular matrix component signaling in cancer. Adv. Drug Deliv. Rev. 97, 28–40. 10.1016/j.addr.2015.10.013 26519775

[B83] Naghavi M. Libby P. Falk E. Casscells S. W. Litovsky S. Rumberger J. (2003). From vulnerable plaque to vulnerable patient: a call for new definitions and risk assessment strategies: part I. Circulation 108 (14), 1664–1672. 10.1161/01.Cir.0000087480.94275.97 14530185

[B84] Newby A. C. (2016). Metalloproteinase production from macrophages - a perfect storm leading to atherosclerotic plaque rupture and myocardial infarction. Exp. Physiol. 101 (11), 1327–1337. 10.1113/ep085567 26969796

[B85] Newman A. A. C. Serbulea V. Baylis R. A. Shankman L. S. Bradley X. Alencar G. F. (2021). Multiple cell types contribute to the atherosclerotic lesion fibrous cap by PDGFRβ and bioenergetic mechanisms. Nat. Metab. 3 (2), 166–181. 10.1038/s42255-020-00338-8 33619382 PMC7905710

[B86] Nguyen M. A. Karunakaran D. Geoffrion M. Cheng H. S. Tandoc K. Perisic Matic L. (2018). Extracellular vesicles secreted by atherogenic macrophages transfer microRNA to inhibit cell migration. Arterioscler. Thromb. Vasc. Biol. 38 (1), 49–63. 10.1161/atvbaha.117.309795 28882869 PMC5884694

[B87] Ohayon J. Finet G. Le Floc'h S. Cloutier G. Gharib A. M. Heroux J. (2014). Biomechanics of atherosclerotic coronary plaque: site, stability and in vivo elasticity modeling. Ann. Biomed. Eng. 42 (2), 269–279. 10.1007/s10439-013-0888-1 24043605 PMC4722860

[B88] Olejarz W. Sadowski K. Radoszkiewicz K. (2023). Extracellular vesicles in atherosclerosis: state of the art. Int. J. Mol. Sci. 25 (1), 388. 10.3390/ijms25010388 38203558 PMC10779125

[B89] Pan H. Xue C. Auerbach B. J. Fan J. Bashore A. C. Cui J. (2020). Single-cell genomics reveals a novel cell state during smooth muscle cell phenotypic switching and potential therapeutic targets for atherosclerosis in mouse and human. Circulation 142 (21), 2060–2075. 10.1161/circulationaha.120.048378 32962412 PMC8104264

[B90] Parma L. Baganha F. Quax P. H. A. de Vries M. R. (2017). Plaque angiogenesis and intraplaque hemorrhage in atherosclerosis. Eur. J. Pharmacol. 816, 107–115. 10.1016/j.ejphar.2017.04.028 28435093

[B91] Patel S. Guo M. K. Abdul Samad M. Howe K. L. (2023). Extracellular vesicles as biomarkers and modulators of atherosclerosis pathogenesis. Front. Cardiovasc Med. 10, 1202187. 10.3389/fcvm.2023.1202187 37304965 PMC10250645

[B92] Patterson M. T. Firulyova M. M. Xu Y. Hillman H. Bishop C. Zhu A. (2023). Trem2 promotes foamy macrophage lipid uptake and survival in atherosclerosis. Nat. Cardiovasc Res. 2 (11), 1015–1031. 10.1038/s44161-023-00354-3 38646596 PMC11031198

[B93] Pedrigi R. M. Mehta V. V. Bovens S. M. Mohri Z. Poulsen C. B. Gsell W. (2016). Influence of shear stress magnitude and direction on atherosclerotic plaque composition. R. Soc. Open Sci. 3 (10), 160588. 10.1098/rsos.160588 27853578 PMC5099003

[B94] Power G. Ferreira-Santos L. Martinez-Lemus L. A. Padilla J. (2024). Integrating molecular and cellular components of endothelial shear stress mechanotransduction. Am. J. Physiol. Heart Circ. Physiol. 327 (4), H989–h1003. 10.1152/ajpheart.00431.2024 39178024 PMC11482243

[B95] Qin X. Zhang K. Qiu J. Wang N. Qu K. Cui Y. (2022). Uptake of oxidative stress-mediated extracellular vesicles by vascular endothelial cells under low magnitude shear stress. Bioact. Mater 9, 397–410. 10.1016/j.bioactmat.2021.10.038 34820579 PMC8586717

[B96] Rao A. Barkley D. França G. S. Yanai I. (2021). Exploring tissue architecture using spatial transcriptomics. Nature 596 (7871), 211–220. 10.1038/s41586-021-03634-9 34381231 PMC8475179

[B97] Ridker P. M. Everett B. M. Thuren T. MacFadyen J. G. Chang W. H. Ballantyne C. (2017). Antiinflammatory therapy with canakinumab for atherosclerotic disease. N. Engl. J. Med. 377 (12), 1119–1131. 10.1056/NEJMoa1707914 28845751

[B98] Ritsvall O. Albinsson S. (2024). Emerging role of YAP/TAZ in vascular mechanotransduction and disease. Microcirculation 31 (4), e12838. 10.1111/micc.12838 38011540

[B99] Roth G. A. Mensah G. A. Johnson C. O. Addolorato G. Ammirati E. Baddour L. M. (2020). Global burden of cardiovascular diseases and risk factors, 1990-2019: update from the GBD 2019 study. J. Am. Coll. Cardiol. 76 (25), 2982–3021. 10.1016/j.jacc.2020.11.010 33309175 PMC7755038

[B100] Roy P. Orecchioni M. Ley K. (2022). How the immune system shapes atherosclerosis: roles of innate and adaptive immunity. Nat. Rev. Immunol. 22 (4), 251–265. 10.1038/s41577-021-00584-1 34389841 PMC10111155

[B101] Saigusa R. Winkels H. Ley K. (2020). T cell subsets and functions in atherosclerosis. Nat. Rev. Cardiol. 17 (7), 387–401. 10.1038/s41569-020-0352-5 32203286 PMC7872210

[B102] Saleem M. Shahzad K. A. Marryum M. Singh S. Zhou Q. Du S. (2024). Exosome-based therapies for inflammatory disorders: a review of recent advances. Stem Cell Res. Ther. 15 (1), 477. 10.1186/s13287-024-04107-2 39695750 PMC11657721

[B103] Samah N. Ugusman A. Hamid A. A. Sulaiman N. Aminuddin A. (2023). Role of matrix metalloproteinase-2 in the development of atherosclerosis among patients with coronary artery disease. Mediat. Inflamm. 2023, 9715114–9715116. 10.1155/2023/9715114 PMC10348858 37457745

[B104] Sawma T. Shaito A. Najm N. Sidani M. Orekhov A. El-Yazbi A. F. (2022). Role of RhoA and Rho-associated kinase in phenotypic switching of vascular smooth muscle cells: implications for vascular function. Atherosclerosis 358, 12–28. 10.1016/j.atherosclerosis.2022.08.012 36049290

[B105] Shellard A. Mayor R. (2021). Durotaxis: the hard path from in vitro to in vivo. Dev. Cell 56 (2), 227–239. 10.1016/j.devcel.2020.11.019 33290722

[B106] Shinge S. A. U. Zhang D. Din A. U. Yu F. Nie Y. (2022). Emerging Piezo1 signaling in inflammation and atherosclerosis; a potential therapeutic target. Int. J. Biol. Sci. 18 (3), 923–941. 10.7150/ijbs.63819 35173527 PMC8771847

[B107] Shu L. X. Cao L. L. Guo X. Wang Z. B. Wang S. Z. (2024). Mechanism of efferocytosis in atherosclerosis. J. Mol. Med. 102 (7), 831–840. 10.1007/s00109-024-02439-3 38727748

[B108] Slenders L. Landsmeer L. P. L. Cui K. Depuydt M. A. C. Verwer M. Mekke J. (2022). Intersecting single-cell transcriptomics and genome-wide association studies identifies crucial cell populations and candidate genes for atherosclerosis. Eur. Heart J. Open 2 (1), oeab043. 10.1093/ehjopen/oeab043 35174364 PMC8841481

[B109] Sluimer J. C. Gasc J. M. van Wanroij J. L. Kisters N. Groeneweg M. Sollewijn Gelpke M. D. (2008). Hypoxia, hypoxia-inducible transcription factor, and macrophages in human atherosclerotic plaques are correlated with intraplaque angiogenesis. J. Am. Coll. Cardiol. 51 (13), 1258–1265. 10.1016/j.jacc.2007.12.025 18371555

[B110] Sopic M. Kararigas G. Devaux Y. Magni P. (2023). Call for participation in the AtheroNET COST action to implement multiomics in atherosclerotic cardiovascular disease research. Eur. Heart J. 44 (24), 2143–2145. 10.1093/eurheartj/ehad153 37042305

[B111] Sutton N. R. Malhotra R. St Hilaire C. Aikawa E. Blumenthal R. S. Gackenbach G. (2023). Molecular mechanisms of vascular health: insights from vascular aging and calcification. Arterioscler. Thromb. Vasc. Biol. 43 (1), 15–29. 10.1161/atvbaha.122.317332 36412195 PMC9793888

[B112] Swanson K. V. Deng M. Ting J. P. (2019). The NLRP3 inflammasome: molecular activation and regulation to therapeutics. Nat. Rev. Immunol. 19 (8), 477–489. 10.1038/s41577-019-0165-0 31036962 PMC7807242

[B113] Swiatlowska P. Sit B. Feng Z. Marhuenda E. Xanthis I. Zingaro S. (2022). Pressure and stiffness sensing together regulate vascular smooth muscle cell phenotype switching. Sci. Adv. 8 (15), eabm3471. 10.1126/sciadv.abm3471 35427166 PMC9012473

[B114] Tabas I. Bornfeldt K. E. (2020). Intracellular and intercellular aspects of macrophage immunometabolism in atherosclerosis. Circ. Res. 126 (9), 1209–1227. 10.1161/circresaha.119.315939 32324504 PMC7392397

[B115] Tall A. R. Bornfeldt K. E. (2023). Inflammasomes and atherosclerosis: a mixed picture. Circ. Res. 132 (11), 1505–1520. 10.1161/circresaha.123.321637 37228237 PMC10213995

[B116] Tang H. Y. Chen A. Q. Zhang H. Gao X. F. Kong X. Q. Zhang J. J. (2022). Vascular smooth muscle cells phenotypic switching in cardiovascular diseases. Cells 11 (24), 4060. 10.3390/cells11244060 36552822 PMC9777337

[B117] Tang H. Zeng R. He E. Zhang I. Ding C. Zhang A. (2022). Piezo-type mechanosensitive ion channel component 1 (Piezo1): a promising therapeutic target and its modulators. J. Med. Chem. 65 (9), 6441–6453. 10.1021/acs.jmedchem.2c00085 35466678

[B118] Tang Y. Yang L. J. Liu H. Song Y. J. Yang Q. Q. Liu Y. (2023). Exosomal miR-27b-3p secreted by visceral adipocytes contributes to endothelial inflammation and atherogenesis. Cell Rep. 42 (1), 111948. 10.1016/j.celrep.2022.111948 36640325

[B119] Thompson W. Papoutsakis E. T. (2023). The role of biomechanical stress in extracellular vesicle formation, composition and activity. Biotechnol. Adv. 66, 108158. 10.1016/j.biotechadv.2023.108158 37105240

[B120] Thorp E. Cui D. Schrijvers D. M. Kuriakose G. Tabas I. (2008). Mertk receptor mutation reduces efferocytosis efficiency and promotes apoptotic cell accumulation and plaque necrosis in atherosclerotic lesions of apoe-/- mice. Arterioscler. Thromb. Vasc. Biol. 28 (8), 1421–1428. 10.1161/atvbaha.108.167197 18451332 PMC2575060

[B121] Tomaniak M. Katagiri Y. Modolo R. de Silva R. Khamis R. Y. Bourantas C. V. (2020). Vulnerable plaques and patients: state-of-the-art. Eur. Heart J. 41 (31), 2997–3004. 10.1093/eurheartj/ehaa227 32402086 PMC8453282

[B122] van Niel G. D'Angelo G. Raposo G. (2018). Shedding light on the cell biology of extracellular vesicles. Nat. Rev. Mol. Cell Biol. 19 (4), 213–228. 10.1038/nrm.2017.125 29339798

[B123] Vandereyken K. Sifrim A. Thienpont B. Voet T. (2023). Methods and applications for single-cell and spatial multi-omics. Nat. Rev. Genet. 24 (8), 494–515. 10.1038/s41576-023-00580-2 36864178 PMC9979144

[B124] Virmani R. Burke A. Farb A. (1998). Coronary risk factors and plaque morphology in men with coronary disease who died suddenly. Eur. Heart J. 19 (5), 678–680. 10.1056/NEJM199705013361802 9716994

[B125] Virmani R. Burke A. P. Farb A. Kolodgie F. D. (2006). Pathology of the vulnerable plaque. J. Am. Coll. Cardiol. 47 (8 Suppl l), C13–C18. 10.1016/j.jacc.2005.10.065 16631505

[B126] Wang K. C. Yeh Y. T. Nguyen P. Limqueco E. Lopez J. Thorossian S. (2016). Flow-dependent YAP/TAZ activities regulate endothelial phenotypes and atherosclerosis. Proc. Natl. Acad. Sci. U. S. A. 113 (41), 11525–11530. 10.1073/pnas.1613121113 27671657 PMC5068257

[B127] Wang L. Chennupati R. Jin Y. J. Li R. Wang S. Günther S. (2020). YAP/TAZ are required to suppress osteogenic differentiation of vascular smooth muscle cells. iScience 23 (12), 101860. 10.1016/j.isci.2020.101860 33319178 PMC7726335

[B128] Wang Y. Shi R. Zhai R. Yang S. Peng T. Zheng F. (2022). Matrix stiffness regulates macrophage polarization in atherosclerosis. Pharmacol. Res. 179, 106236. 10.1016/j.phrs.2022.106236 35483516

[B129] Wang Z. Su J. Gong F. Xue L. Su Z. (2022). The impaired mechanism and facilitated therapies of efferocytosis in atherosclerosis. J. Cardiovasc Pharmacol. 80 (3), 407–416. 10.1097/fjc.0000000000001311 35853202

[B130] Wang X. Shen Y. Shang M. Liu X. Munn L. L. (2023). Endothelial mechanobiology in atherosclerosis. Cardiovasc Res. 119 (8), 1656–1675. 10.1093/cvr/cvad076 37163659 PMC10325702

[B131] Wang J. Wu Q. Wang X. Liu H. Chen M. Xu L. (2024). Targeting macrophage phenotypes and metabolism as novel therapeutic approaches in atherosclerosis and related cardiovascular diseases. Curr. Atheroscler. Rep. 26 (10), 573–588. 10.1007/s11883-024-01229-z 39133247 PMC11392985

[B132] Wang S. Wang X. Lv Y. Zhang Z. He T. Hao X. (2024). M2 macrophage-derived exosomes inhibit atherosclerosis progression by regulating the proliferation, migration, and phenotypic transformation of smooth muscle cells. Front. Biosci. (Landmark Ed) 29 (8), 288. 10.31083/j.fbl2908288 39206919

[B133] Weiss L. Macleod H. Maguire P. B. (2025). Platelet-derived extracellular vesicles in cardiovascular disease and treatment - from maintaining homeostasis to targeted drug delivery. Curr. Opin. Hematol. 32 (1), 4–13. 10.1097/moh.0000000000000845 39377239 PMC11620325

[B134] Welsh J. A. Goberdhan D. C. I. O'Driscoll L. Buzas E. I. Blenkiron C. Bussolati B. (2024). Minimal information for studies of extracellular vesicles (MISEV2023): from basic to advanced approaches. J. Extracell. Vesicles 13 (2), e12404. 10.1002/jev2.12404 38326288 PMC10850029

[B135] Winkels H. Ehinger E. Vassallo M. Buscher K. Dinh H. Q. Kobiyama K. (2018). Atlas of the immune cell repertoire in mouse atherosclerosis defined by single-cell RNA-sequencing and mass cytometry. Circ. Res. 122 (12), 1675–1688. 10.1161/circresaha.117.312513 29545366 PMC5993603

[B136] Wirka R. C. Wagh D. Paik D. T. Pjanic M. Nguyen T. Miller C. L. (2019). Atheroprotective roles of smooth muscle cell phenotypic modulation and the TCF21 disease gene as revealed by single-cell analysis. Nat. Med. 25 (8), 1280–1289. 10.1038/s41591-019-0512-5 31359001 PMC7274198

[B137] Wolf D. Ley K. (2019). Immunity and inflammation in atherosclerosis. Circ. Res. 124 (2), 315–327. 10.1161/circresaha.118.313591 30653442 PMC6342482

[B138] Xie L. Chen J. Hu H. Zhu Y. Wang X. Zhou S. (2024). Engineered M2 macrophage-derived extracellular vesicles with platelet membrane fusion for targeted therapy of atherosclerosis. Bioact. Mater 35, 447–460. 10.1016/j.bioactmat.2024.02.015 38390527 PMC10881364

[B139] Yang W. Zou B. Hou Y. Yan W. Chen T. Qu S. (2019). Extracellular vesicles in vascular calcification. Clin. Chim. Acta 499, 118–122. 10.1016/j.cca.2019.09.002 31493375

[B140] Yin L. Wang X. Xiong N. Xiong J. Liu Q. Li H. (2026). The role of the NF-κB signaling pathway in atherosclerotic plaque rupture and targeted therapeutic strategies. Biomedicines 14 (1), 201. 10.3390/biomedicines14010201 41595734 PMC12838933

[B141] Yurdagul A. Jr. Doran A. C. Cai B. Fredman G. Tabas I. A. (2017). Mechanisms and consequences of defective efferocytosis in atherosclerosis. Front. Cardiovasc Med. 4, 86. 10.3389/fcvm.2017.00086 29379788 PMC5770804

[B142] Zeng Y. Cui X. Li H. Wang Y. Cheng M. Zhang X. (2024). Extracellular vesicles originating from the mechanical microenvironment in the pathogenesis and applications for cardiovascular diseases. Regen. Ther. 26, 1069–1077. 10.1016/j.reth.2024.10.012 39582802 PMC11585476

[B143] Zernecke A. Winkels H. Cochain C. Williams J. W. Wolf D. Soehnlein O. (2020). Meta-analysis of leukocyte diversity in atherosclerotic mouse aortas. Circ. Res. 127 (3), 402–426. 10.1161/circresaha.120.316903 32673538 PMC7371244

[B144] Zernecke A. Erhard F. Weinberger T. Schulz C. Ley K. Saliba A. E. (2023). Integrated single-cell analysis-based classification of vascular mononuclear phagocytes in mouse and human atherosclerosis. Cardiovasc Res. 119 (8), 1676–1689. 10.1093/cvr/cvac161 36190844 PMC10325698

[B145] Zhai K.-R. Zhao P.-L. Wang Z.-H. Feng H.-M. Li Z.-Q. Huang H.-R. (2026). Caveolin-1 drives immunosuppression in esophageal squamous cell carcinoma by enhancing exosome secretion and macrophage M2 polarization via inhibition of MVB autophagic degradation. J. Transl. Med. 24 (1), 313. 10.1186/s12967-026-07800-3 41639737 PMC12958683

[B146] Zhang Y. Wang Y. Zhou D. Zhang L. S. Deng F. X. Shu S. (2019). Angiotensin II deteriorates advanced atherosclerosis by promoting MerTK cleavage and impairing efferocytosis through the AT(1)R/ROS/p38 MAPK/ADAM17 pathway. Am. J. Physiol. Cell Physiol. 317 (4), C776–c787. 10.1152/ajpcell.00145.2019 31390228

[B147] Zhang Y. G. Song Y. Guo X. L. Miao R. Y. Fu Y. Q. Miao C. F. (2019). Exosomes derived from oxLDL-stimulated macrophages induce neutrophil extracellular traps to drive atherosclerosis. Cell Cycle 18 (20), 2674–2684. 10.1080/15384101.2019.1654797 31416388 PMC6773244

[B148] Zheng Q. Zou Y. Teng P. Chen Z. Wu Y. Dai X. (2022). Mechanosensitive channel PIEZO1 senses shear force to induce KLF2/4 expression via CaMKII/MEKK3/ERK5 axis in endothelial cells. Cells 11 (14), 2191. 10.3390/cells11142191 35883633 PMC9317998

[B149] Zhou M. Yu Y. Chen R. Liu X. Hu Y. Ma Z. (2023). Wall shear stress and its role in atherosclerosis. Front. Cardiovasc Med. 10, 1083547. 10.3389/fcvm.2023.1083547 37077735 PMC10106633

[B150] Zhu J. Liu B. Wang Z. Wang D. Ni H. Zhang L. (2019). Exosomes from nicotine-stimulated macrophages accelerate atherosclerosis through miR-21-3p/PTEN-mediated VSMC migration and proliferation. Theranostics 9 (23), 6901–6919. 10.7150/thno.37357 31660076 PMC6815950

[B151] Zhu Y. Chen J. Chen C. Tang R. Xu J. Shi S. (2025). Deciphering mechanical cues in the microenvironment: from non-malignant settings to tumor progression. Biomark. Res. 13 (1), 11. 10.1186/s40364-025-00727-9 39849659 PMC11755887

